# The prediction accuracy of dynamic mixed-effects models in clustered data

**DOI:** 10.1186/s13040-016-0084-6

**Published:** 2016-01-27

**Authors:** Brian S. Finkelman, Benjamin French, Stephen E. Kimmel

**Affiliations:** Center for Clinical Epidemiology and Biostatistics, Department of Biostatistics and Epidemiology, Perelman School of Medicine, University of Pennsylvania, Philadelphia, PA USA; Center for Therapeutic Effectiveness Research, Perelman School of Medicine, University of Pennsylvania, Philadelphia, PA USA; Department of Medicine, Cardiovascular Division, Perelman School of Medicine, University of Pennsylvania, Philadelphia, PA USA

**Keywords:** Dynamic modeling, Bayesian statistics, Mixed-effects models, Prediction, Clustered data, Generalizability

## Abstract

**Background:**

Clinical prediction models often fail to generalize in the context of clustered data, because most models fail to account for heterogeneity in outcome values and covariate effects across clusters. Furthermore, standard approaches for modeling clustered data, including generalized linear mixed-effects models, would not be expected to provide accurate predictions in novel clusters, because such predictions are typically based on the hypothetical mean cluster. We hypothesized that dynamic mixed-effects models, which incorporate data from previous predictions to refine the model for future predictions, would allow for cluster-specific predictions in novel clusters as the model is updated over time, thus improving overall model generalizability.

**Results:**

We quantified the potential gains in prediction accuracy from using a dynamic modeling strategy in a simulation study. Furthermore, because clinical prediction models in the context of clustered data often involve outcomes that are dependent on patient volume, we examined whether using dynamic mixed-effects models would be robust to misspecification of the volume-outcome relationship. Our results indicated that dynamic mixed-effects models led to substantial improvements in prediction accuracy in clustered populations over a broad range of conditions, and were uniformly superior to static models. In addition, dynamic mixed-effects models were particularly robust to misspecification of the volume-outcome relationship and to variation in the frequency of model updating. The extent of the improvement in prediction accuracy that was observed with dynamic mixed-effects models depended on the relative impact of fixed and random effects on the outcome as well as the degree of misspecification of model fixed effects.

**Conclusions:**

Dynamic mixed-effects models led to substantial improvements in prediction model accuracy across a broad range of simulated conditions. Therefore, dynamic mixed-effects models could be a useful alternative to standard static models for improving the generalizability of clinical prediction models in the setting of clustered data, and, thus, well worth the logistical challenges that may accompany their implementation in practice.

**Electronic supplementary material:**

The online version of this article (doi:10.1186/s13040-016-0084-6) contains supplementary material, which is available to authorized users.

## Background

Despite the widespread adoption of prediction models in clinical research and medical practice, there are often major concerns about model generalizability across different populations and clinical settings. For instance, the EuroSCORE model, which was developed in European populations to predict 30-day mortality in patients undergoing cardiac surgery, failed to generalize to Australian surgical patients [1], and, even within the European population, proved inaccurate over time, over-predicting risk in contemporary practice [2]. In another example, a clinical prediction rule for predicting deep vein thrombosis performed well in the secondary referral patient population in which it was developed, but failed to generalize to a primary care setting [3]. This problem is likely even more widespread than what has been directly documented in the literature because of the many clinical outcomes that are known to vary substantially across clinical sites, including readmission after hospitalization for heart failure [4], mortality following surgery for colorectal cancer [5], false-positive results from mammographic screening [6], graft failure after liver transplantation [7], and medication adherence rates among diabetes patients [8]. However, despite the high prevalence of such problems, relatively little research has been done to develop general approaches for improving model performance in the context of clustered, heterogeneous populations. Notably, established methods for reducing overfitting, such as Bayesian model averaging [9], bootstrap aggregation or bagging [10], and cross-validation [11], would not be expected to improve model generalizability in this context, because they are unable to test the model on samples from a different empirical distribution than the derivation dataset, which is generally composed of data from a small number of clusters within a larger clustered population.

One standard approach to modeling clustered data is with generalized linear mixed-effects models (GLMM), which use random effects to parameterize heterogeneity in effects across clusters and induce a within-cluster correlation structure [12]. Although GLMMs are theoretically capable of producing cluster-specific predictions, they would not be expected to improve overall model generalizability across a clustered population, because predictions on novel clusters (i.e. those that are not included in the original data sample) are still based on the hypothetical mean cluster [13]. As a result, any improvement in prediction accuracy that results from using mixed-effects models is generally because of shrinkage effects, rather than incorporating knowledge about cluster-specific differences. This limitation may explain why GLMMs are not used more frequently for clinical prediction models.

However, this limitation of standard GLMMs may be alleviated if they are estimated in a dynamic fashion. Dynamic prediction models have been recently proposed as a method to improve the calibration of prediction models over time. In dynamic prediction modeling, predictions are made on individuals using the best available model at that time. Then, as the outcome data from previous predictions become available, they can be incorporated into the data sample and used to update or refine the model. Model updating can be performed in an online fashion through the continual adjustment of Bayesian priors or by re-estimating the model using all of the available data in either a Bayesian or frequentist approach [2, 14]. This method has been successfully demonstrated in empirical examples [2, 14]. However, to our knowledge, this approach has not previously been extended to prediction models in the context of clustered data, for which it might be expected to improve model generalizability. In particular, if a dynamic modeling framework were applied to mixed-effects models, novel clusters would be converted into existing clusters within the data sample over time, allowing for predictions that account for cluster-specific differences. Thus, with dynamic mixed-effects models, model generalizability can be improved over time as the model is used and updated over an increasing number of unique clusters. Furthermore, previous research has not studied the impact of model misspecification or updating frequency on the accuracy of dynamic prediction models. These latter questions, in particular, are not easily addressed analytically and require direct testing via simulation.

In addition, many important clinical outcomes in the setting of clustered data show a relationship between the outcome and cluster size, which is often referred to in the clinical literature as ‘volume.’ For instance, it is well established that mortality following major surgery is inversely related to the volume of patients receiving a given surgery at a particular hospital [15]. This relationship has held true for many different specific areas of surgery, as well, including thoracic [16], oncologic [17], and endovascular surgery [18]. Hospital volume is also an important predictor of mortality following hospitalization for myocardial infarction [19], mortality following inpatient mechanical ventilation [20], and mortality following hospitalization for severe sepsis [21], among other outcomes. Because many specific cases of prediction models in the context of clustered data would be expected to have this volume-outcome relationship, it is important to determine the robustness of the dynamic mixed-effects model approach to misspecification of this association. Furthermore, while many other cluster-specific effects would be expected to be easily accommodated by random intercepts and slopes in dynamic mixed-effects models, the effect of volume could theoretically behave differently because it is directly related to the probability of observing the data responsible for the updating process. In other words, predictions at smaller clusters could become biased because the predictions are too heavily shrunk toward the predictions at larger clusters, which make up the preponderance of the data. As a result, the robustness of the dynamic approach to misspecification of the volume-outcome relationship needs to be specifically assessed.

In this paper, we sought to quantify the potential improvement in prediction accuracy from dynamic mixed-effects models in the context of clustered data via a simulation study. We also examined whether using dynamic mixed-effects models would be robust to misspecification of the volume-outcome relationship, misspecification of model fixed effects, and variable frequencies of model updating. The results of these simulations demonstrate the general utility of dynamic mixed-effects models for producing more generalizable clinical prediction models in the setting of clustered data, and provide motivation for further research toward solving the logistical and analytical challenges that may accompany this approach in practice.

## Methods

### Dynamic mixed-effects models

GLMMs account for clustering in the outcome by treating some model parameters as random, rather than fixed, across the population. These models typically follow the form:

1 $$ g\left(\mathrm{E}\left[{Y}_{ij}\Big|{X}_{ij},{b}_i\right]\right)={X}_{ij}\beta +{Z}_i{b}_i, $$

in which the link function *g*(∙) relates the average outcome *Y*_*ij*_ for individual *j* in cluster *i* to the observed covariate design matrices *X*_*ij*_ and *Z*_*i*_ through a vector of fixed effects *β* and a vector of random effects *b*_*i*_, respectively [12]. Random effects are typically modeled parametrically as $\mathcal{N}\left(0,G\right)$, where *G* is the variance-covariance matrix of the random effects. Use of this parametric structure for the random effects is typically more efficient than cluster-level fixed effects, making it especially useful in settings where there are a large number of clusters.

Mixed-effects models can be estimated using either a frequentist or Bayesian approach. In the context of Bayesian linear mixed-effects (BLME) models, prior distributions for *β* and *b*_*i*_ —as well as the variance of the residual error conditional on the random effects, *σ*^2^ —are fully specified and used to estimate posterior distributions based on available data. The parametric structure of the random effects is specified as hyperpriors on the distribution of *b*_*i*_. Thus, using our previous notation, the posterior distribution of model parameters can be estimated conditional on the observed data as:

2 $$ p\left(\beta, {b}_i,{\sigma}^2\Big|{Y}_{ij}\right)\propto p\left({Y}_{ij}\Big|\beta, {b}_i,{\sigma}^2\right)p\left(\beta, {b}_i,{\sigma}^2\right), $$

for which *p*(*β*, *b*_*i*_, *σ*^2^) is the prior distribution of all model parameters and *p*(*Y*_*ij*_|*β*, *b*_*i*_, *σ*^2^) is the likelihood of the observed data given the model. Depending on the specific application and the availability of prior information, prior distributions can be specified as informative or non-informative. Use of non-informative priors is reflective of a typical scenario for the initial development of a prediction model, when most researchers would want to “let the data speak for themselves.”

We refer to the above models as ‘static’ models, because once they are estimated in the derivation dataset, which we call the ‘training sample,’ the resulting model is used to make out-of-sample predictions on the remainder of the population, which we call the ‘testing sample,’ without any further refinement or adjustment. By contrast, dynamic models are designed to capture data from out-of-sample predictions to update the model for future predictions. Thus, the number of observations in the training sample grows over time *t*, such that:

3 $$ {\displaystyle {\sum}_{i=1}^{m(t)}}{N}_i(t)\le {\displaystyle {\sum}_{i=1}^{m\left(t+\Delta t\right)}}{N}_i\left(t+\Delta t\right), $$

for which *N*_*i*_(*t*) is the number of patients in the training sample for cluster *i* at time *t*, and *m*(*t*) is the number of clusters in the training sample at time *t*. Also implied here is that the number of clusters in the training sample is growing over time, or *m*(*t*) ≤ *m*(*t* + Δ*t*). Static models are therefore a subset of dynamic models for which *N*_*i*_(*t*) = *N*_*i*_(*t* + Δ*t*) and *m*(*t*) = *m*(*t* + Δ*t*) for all *t*. Furthermore, the quantity of data over time is really the only difference between the two types of models, and, at any time *t*, the dynamic model is equivalent to the static model that would have been produced if the original training sample were the same as the training sample at time *t*. Note that the model priors are not changing over time in our approach; however, accounting for previous data in dynamic priors could be an alternative approach to implementing a dynamic prediction model [14].

Combining a dynamic modeling approach with generalized mixed-effects models would therefore be expected to allow a single prediction model to calibrate to local conditions, by incorporating novel clusters into the data sample used for model estimation over time. In essence, many predictions that would have been made based on the hypothetical mean cluster (*b*_*i*_ = 0) with static models can be made using cluster-specific random effects with dynamic models, and the extent of the improvement in prediction accuracy from dynamic prediction models should depend on how quickly these cluster-specific random effects can be estimated. Additionally, as with any mixed-effects model, predictions at individual clusters are able to borrow strength from data at other clusters to avoid the overfitting that might occur if separate models were fit at each cluster.

### Simulation study

In our simulation, we aimed to develop and assess the accuracy of a model to predict a hypothetical clinical outcome for individual patients, who are clustered within clinics. The outcome *Y*_*ij*_*—*which represented a hypothetical normally distributed, continuous clinical outcome for patient *j* at clinic *i—*was dependent on *X*_1*ij*_, a known patient-level predictor; *X*_2*ij*_, an unknown patient-level predictor; and *N*_*i*_, the size of the clinic. Note that *X*_1*ij*_ and *X*_2*ij*_ can also be interpreted as linear combinations of important predictors, rather than just a single predictor. Clustering of the outcome was induced by a clinic-level random intercept *b*_0*i*_ and random slopes *b*_1*i*_ and *b*_2*i*_. From 500 total clinics in the population, 20 were randomly selected as the training sample. Using the training sample, we fit both dynamic and static versions of models with fixed effects only, as well as those with random intercepts and random slopes. These models were then assessed in the remaining clinics in the population, which constituted the testing sample. For each combination of parameter values, the simulation was run 1,000 times to estimate the degree of variability in the results. All simulations were performed using R 3.1.1 [22].

### Data-generating process

For all simulations, we first generated a population of 500 clinics, each with *N*_*i*_ patients, with:

4 $$ {N}_i \sim \left\lceil \exp \left(\mathcal{N}\left({\mu}_N,{\sigma}_N^2\right)\right)\right\rceil $$

The log-normal distribution ensured that there were a large number of smaller clinics, with a small number of very large clinics. The value for *μ*_*N*_, for which exp (*μ*_*N*_) was equivalent to the median clinic size, was fixed at ln(65), while the value for *σ*_*N*_ was fixed at ln(2), in order to ensure a range of clinic sizes of approximately 10 to 500 patients. Note that *N*_*i*_ refers to the number of patients at a clinic for whom predictions will be made; patients at a given clinic who are not candidates for prediction do not matter for purposes of this simulation.

Next, clinic-level random intercepts and slopes were generated from a multivariate normal distribution:

5 $$ \left\{{b}_{0i},{b}_{1i},{b}_{2i}\right\} \sim \mathcal{N}\left(0,\mathrm{T}\right), $$

for which *b*_0*i*_ was the random intercept, *b*_1*i*_ was the random slope for *X*_1*ij*_, *b*_2*i*_ was the random slope for *X*_2*ij*_, and the variance-covariance matrix was:

6 $$ \mathrm{T}=\left[\begin{array}{ccc}\hfill {\tau}_0^2\hfill & \hfill \rho {\tau}_1{\tau}_0\hfill & \hfill \rho {\tau}_2{\tau}_0\hfill \\ {}\hfill \rho {\tau}_0{\tau}_1\hfill & \hfill {\tau}_1^2\hfill & \hfill \rho {\tau}_2{\tau}_1\hfill \\ {}\hfill \rho {\tau}_0{\tau}_2\hfill & \hfill \rho {\tau}_1{\tau}_2\hfill & \hfill {\tau}_2^2\hfill \end{array}\right] $$

The correlation between the random intercept and random slopes, *ρ*, was fixed at a moderate value of 0.3, which was felt to be similar to what might be observed in practice. However, sensitivity analyses demonstrated that the results were insensitive to increases or decreases in the value of the correlation (data not shown). Additionally, we determined that having the correlation between the random slopes differ from the correlation between the random intercept and random slopes did not have a substantial impact on the results (data not shown), so the same value for all correlations was used to improve model simplicity.

After clinic-level random effects were generated, patient-level variables were generated. First, *X*_1*ij*_ and *X*_2*ij*_ were generated as $\mathcal{N}\left(0,1\right)$ variables. The variance for these variables was fixed at 1 for all parameter combinations to provide a reference point for easier interpretation of the values of other parameters. We varied *τ*_0_^2^ and *τ*_1_^2^ to determine the impact of different relative strengths of clinic-level heterogeneities, compared to patient-level factors.

The outcome *Y*_*ij*_ was then generated as:

7 $$ {Y}_{ij}={b}_{0i}+\left({\beta}_1+{b}_{1i}\right){X}_{1ij}+\left({\beta}_2+{b}_{2i}\right){X}_{2ij}+\gamma f\left({N}_i\right)+{\epsilon}_{ij}, $$

for which *ϵ*_*ij*_ were independent errors distributed as $\mathcal{N}\left(0,{\sigma}_{\epsilon}^2\right)$. The value of *σ*_*ϵ*_^2^ was calculated as:

8 $$ {\sigma}_{\epsilon}^2=\frac{\alpha }{1-\alpha}\mathrm{V}\mathrm{a}\mathrm{r}\left({b}_{0i}+\left({\beta}_1+{b}_{1i}\right){X}_{1ij}+\left({\beta}_2+{b}_{2i}\right){X}_{2ij}+\gamma f\left({N}_i\right)\right), $$

with a value of *α* = *σ*_*ϵ*_^2^/*σ*_*Y*_^2^ = 0.2 chosen so that the variance of the residual error terms was equal to 20 % of the total variance in *Y*_*ij*_, denoted by *σ*_*Y*_^2^. This value was thought to be reflective of a typical high-quality clinical prediction model developed by rigorous methods, where the majority of the variance is explained by the model. The value of *α* was varied in sensitivity analyses to ensure that the results of the simulation were not dependent on the value of this parameter. Clinic size was associated with the outcome through the function *f*(∙), with:

9 $$ f\left({N}_i\right)=\Omega \left( \ln \left({N}_i\right)-\mathrm{mean}\left( \ln \left({N}_i\right)\right)\right), $$

for which Ω was a scaling factor such that $f\left({N}_i\right) \sim \mathcal{N}\left(0,1\right)$. The value for *β*_1_ was fixed at one across all simulations, so that *β*_2_ and *γ* gain the interpretation of the impact of *X*_2*ij*_ and clinic size on the outcome, respectively, relative to the impact of *X*_1*ij*_. Note that the overall intercept across all clinics, *β*_0_, was defined as equal to 0 and is thus not included in Equation 7.

### Parameter values

The main parameters that were varied for our simulation were *τ*_0_^2^ and *τ*_1_^2^, which controlled the relative impact of patient-level factors and clinic-level heterogeneities on the outcome. Three values of each parameter were examined—0.5, 1, and 2 for *τ*_0_^2^, and 0, 0.25, and 0.5 for *τ*_1_^2^ —for a total of 9 main parameter combinations. The values of these parameters can be interpreted relative to the size of the variance in *X*_1*ij*_, which was fixed at 1. Additionally, *β*_2_ and *γ* were fixed at zero for these main parameter combinations, so that the effects of unknown patient-level factors and clinic size on the results could be examined in isolation. When *β*_2_ was equal to zero, *τ*_2_^2^ was also set equal to zero, so that there was no effect of *X*_2*ij*_ on *Y*_*ij*_; when *β*_2_ was not equal to zero, *τ*_2_^2^ was set to be equal to *τ*_1_^2^. We considered *τ*_0_^2^ = 1, *τ*_1_^2^ = 0.25, *β*_2_ = 0, and *γ* = 0 to be the ‘base’ parameter combination, and sensitivity analyses for individual parameters were based on this combination of parameter values. For reference, in the base parameter combination, *τ*_0_/*σ*_*ϵ*_ ≈ 4/3 and *τ*_1_/*σ*_*ϵ*_ ≈ 2/3.

Next, we separately assessed the impact of non-zero values for *β*_2_ and *γ*. Specifically, we examined values of $\sqrt{0.5}$, 1, and $\sqrt{2}$ for both parameters. These values were selected for greater interpretability, as the relative contribution of *X*_2*ij*_ and *f*(*N*_*i*_) to the total variance in *Y*_*ij*_ was proportional to *β*_2_^2^ and *γ*^2^, respectively. Thus, for example, when ${\beta}_2=\sqrt{2}$, *X*_2*ij*_ contributed twice as much to the variance in *Y*_*ij*_ as did *X*_1*ij*_. This set of parameter values likely covers the full range of what could reasonably be expected in practice, assuming that prediction models would still be developed using rigorous methods and high quality data. However, more extreme values of *β*_2_ were also examined in sensitivity analyses. For this set of parameter combinations, *τ*_0_^2^ and *τ*_1_^2^ were fixed at their base values.

Finally, we assessed the impact of varying update intervals in an attempt to reflect longer time lags between predictions and the occurrence of the outcome, which might take place in certain clinical scenarios, such as those with survival-type outcomes. We examined values of 250, 500, 1,000, and 5,000 for *θ* —where *θ* is the number of predictions made between cycles of updating for dynamic models, and 0.8 * *θ* is the expected number of new subjects incorporated into the dynamic models at each iteration, as described below. We used *θ* = 500 as its base value for all previously described parameter combinations.

### Prediction models

We randomly selected 20 clinics—stratified by clinic-size quintile, *N*_i_^*^ —for the training sample, mimicking a large multi-center cohort that might be used to develop a clinical prediction model in practice. We selected 6 clinics from each of the bottom two quintiles, three clinics from each of the next two quintiles, and two clinics from the upper quintile. We then developed three prediction models in the training sample:

1. A linear model, *β*_1_*X*_1*ij*_;
2. A BLME model with a random intercept, *b*_0*i*_ + *β*_1_*X*_1*ij*_;
3. A second BLME model with a random intercept and slope, *b*_0*i*_ + (*β*_1_ + *b*_1*i*_)*X*_1*ij*_.

BLME models were fit using restricted maximum likelihood, with non-informative flat priors for the fixed effects and a non-informative prior for the random effects covariance matrix based on the Wishart distribution. Estimation of BLME models was accomplished using the blme extension package in R [23]. Additionally, for simulations when *γ* ≠ 0, we also constructed versions of the above models that included *N*_i_^*^ as a categorical fixed effect, because it was felt that *N*_i_^*^ would be more likely to be observable than *f*(*N*_*i*_) in practice.

All three models were assessed in the testing sample both as dynamic and static models. Note that the static linear model is meant to reflect the typical prediction model that would be developed and used in practice. Dynamic modeling was achieved by making predictions on *θ* patients, incorporating outcome data on those individuals back into the training sample, re-estimating the models, and then making predictions on the next *θ* patients. This algorithm was repeated until predictions had been made on all patients in the testing sample. For BLME models, this was equivalent to adding new data, and did not affect the model priors. The order of predictions was random across the entire testing sample, and each individual had an 80 % chance to have their outcome data incorporated into the training sample for future model updates. We chose 80 % because it realistically allows for missing outcome data; this is reflective of missing outcome data that might occur when utilizing a dynamic prediction modeling scheme in practice, where patients might be lost to follow-up before their outcomes are observed. Note that in this set-up, the expected number of new subjects incorporated into the dynamic model at each iteration is 80 % of the value of *θ*.

### Assessment of model calibration

Accuracy of prediction models was based on model calibration, which was assessed as mean absolute error (MAE) [24]. MAE was calculated as:

10 $$ \mathrm{M}\mathrm{A}\mathrm{E}={\phi}_{model}=\frac{1}{n}{\displaystyle \sum}\left|{\widehat{Y}}_{ij}-{Y}_{ij}\right|, $$

for which *n* was the total number of individuals in the training sample. To improve the interpretability of the results, we constructed a metric, the ‘relative improvement’ (RI) in MAE, for each model, which was calculated as:

11 $$ \mathrm{R}\mathrm{I}=\frac{\phi_0-{\phi}_{model}}{\phi_0-{\phi}_1}, $$

where *ϕ*_0_ refers to the MAE for the intercept-only model, as fit in the training sample, and *ϕ*_1_ refers to the MAE for the ‘true’ model, which was considered to be the model in Equation 7, minus the residual error term, *ϵ*_*ij*_. Thus, the RI will typically range from 0 to 1 and can be interpreted as the improvement of the current model over the intercept-only model, relative to the improvement that would have been seen with the true model. Negative values for RI indicate that the given model is worse than predicting the average value in all individuals. Thus, the RI for a given model is analogous to the relative utility metric proposed by Baker [25], except in the context of model calibration and without the decision-theoretic weighting scheme. Furthermore, because the MAE of all of the models contain the same residual error, *ϵ*_*ij*_, this term is factored out of the RI, giving the metric the advantageous feature of being relatively insensitive to changes in the magnitude of *σ*_*ϵ*_^2^.

## Results

### Population characteristics

There were 41,576 (SD 1,465) patients in the total simulated population, on average, with 1,276 (SD 118) patients in the training sample. Within a given simulation, clinics ranged in size from 9 to 549 patients, on average. The median clinic had 66 patients, and 67 % of patients were in clinics in the top two quintiles of clinic size. Clinics in the smallest quintile of clinic size had between 9 and 36 patients, on average, while those in the largest quintile had 117 or more patients, on average. A visualization of the effect of varying *τ*_0_^2^ and *τ*_1_^2^ on clinic-level clustering can be seen in Additional file 1: Figure S1.

### Main parameter results

**Fig. 1:**
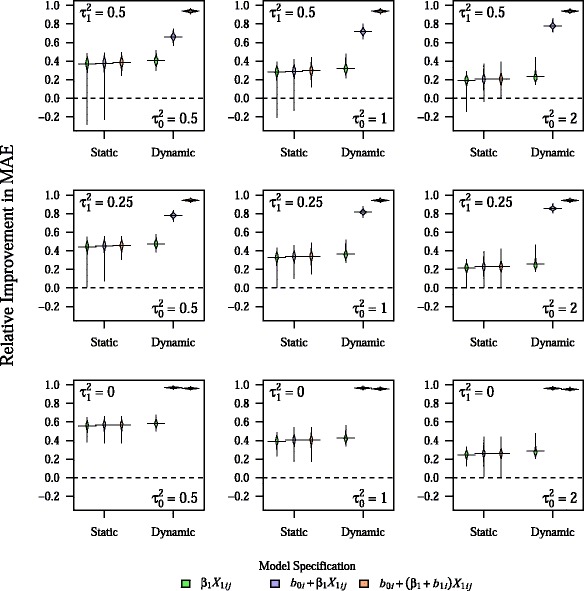
Relative improvement in MAE for both dynamic and static models across all main parameter combinations. Plots show the density of values for relative improvement in MAE across 1,000 simulations, with horizontal bars representing the mean value. All other parameters are fixed at their base values. The center figure represents the base parameter combination

As can be seen in Table 1, the prediction models were very accurate in the training sample, with the RI in the training sample ranging from 24 to 101 %, depending on the model and parameter combination. In particular, RI in the training sample was uniformly 101 % for the BLME model with both a random intercept and random slope, indicating overfitting. Although the addition of random effects led to dramatic improvements in the model accuracy in the training sample, they led to virtually no improvement in the accuracy of predictions as assessed in the testing sample, with a mean RI of 33 to 34 % for all static models for the base parameter combination. In contrast, use of dynamic modeling led to dramatic improvements in RI for both BLME models, across all main parameter combinations tested, with RI values generally in excess of 70 % (Fig. 1).

**Table 1:** Mean RI for static models in training sample for all main parameter combinations

*τ*_1_^2^	Model	*τ*_0_^2^ = 0.5	*τ*_0_^2^ = 1	*τ*_0_^2^ = 2
	*β*_1_ *X*_1_	0.424 (0.121)	0.337 (0.114)	0.244 (0.101)
0.5	*b*_0*i*_ + *β*_1_ *X*_1_	0.714 (0.099)	0.767 (0.084)	0.826 (0.068)
	*b*_0*i*_ + (*β*_1_ + *b*_1*i*_)*X*_1_	1.014 (0.006)	1.014 (0.005)	1.014 (0.006)
	*β*_1_ *X*_1_	0.496 (0.105)	0.382 (0.107)	0.267 (0.097)
0.25	*b*_0*i*_ + *β*_1_ *X*_1_	0.827 (0.067)	0.863 (0.056)	0.902 (0.044)
	*b*_0*i*_ + (*β*_1_ + *b*_1*i*_)*X*_1_	1.014 (0.005)	1.013 (0.005)	1.013 (0.005)
	*β*_1_ *X*_1_	0.604 (0.098)	0.445 (0.106)	0.297 (0.097)
0	*b*_0*i*_ + *β*_1_ *X*_1_	1.007 (0.003)	1.007 (0.003)	1.007 (0.004)
	*b*_0*i*_ + (*β*_1_ + *b*_1*i*_)*X*_1_	1.013 (0.004)	1.013 (0.005)	1.013 (0.005)

Results presented as mean (SD) for 1,000 simulations

**Fig. 2:**
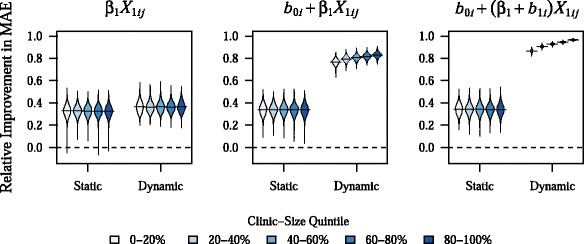
Relative improvement in MAE by clinic-size quintile. Plots show the density of values for relative improvement in MAE across 1,000 simulations, with horizontal bars representing the mean value. These results are for the base parameter combination

**Fig. 3:**
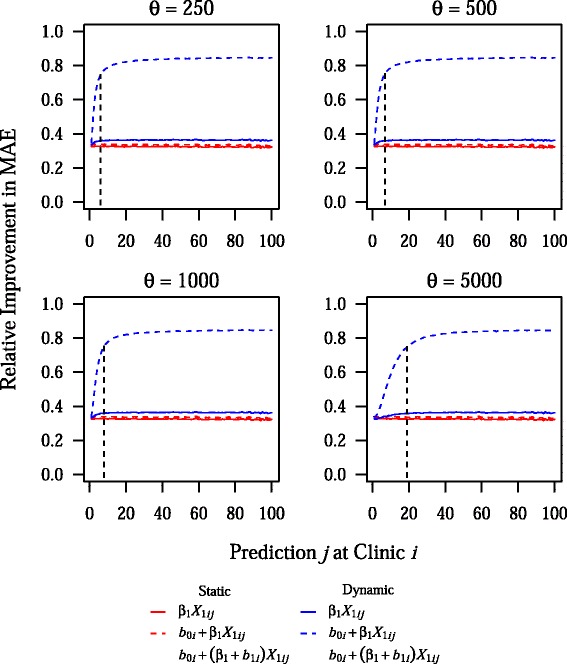
Effect of the update interval on the rate of improvement in prediction accuracy at a given clinic. This plot shows the mean relative improvement in MAE for prediction *j* at clinic *i*, across 1,000 simulations for different values of the update interval, *θ*. Vertical dashed and dotted lines indicate the point at which 80 % of the total gains in prediction accuracy have been achieved for the dynamic BLME model with a random intercept and the dynamic BLME model with a random intercept and random slope, respectively. Note that the base value of *θ* is 500, and all other parameters are fixed at their base values

As can be seen in Fig. 2, gains in prediction accuracy from dynamic mixed-effects models were seen across all clinic-size quintiles, although the greatest improvement was seen in the largest clinics. This pattern likely reflects the fact that improvements from dynamic modeling were seen relatively rapidly, with approximately 80 % of the total gains in predictive performance for the dynamic BLME models occurring on average after about 7 and 9 predictions at a given clinic for the model with a random intercept and the model with both a random intercept and random slope, respectively (Fig. 3). Because there were 480 clinics in the testing sample and the model was updated after every 500 predictions, model updates occurred after almost every prediction, especially at smaller clinics. The rate of improvement in predictive accuracy was somewhat sensitive to changes in *σ*_*ϵ*_^2^, however, with 80 % of the total gains in predictive performance for the dynamic BLME model with both a random intercept and slope occurring after about 17 predictions at a given clinic, on average, when the residual error was equal to 50 % of the overall variance in *Y*_*ij*_ (Additional file 1: Figure S2). Noticeable decreases in RI values for dynamic BLME models were seen only at very extreme values for *σ*_*ϵ*_^2^, such as when the residual error was equal to 80 % of the total variance in *Y*_*ij*_ (Additional file 1: Figure S3). However, even at this extreme and likely unrealistic parameter value, dynamic BLME models outperformed static models, with RI values for the former in excess of 70 %.

### Effect of model misspecification

**Fig. 4:**
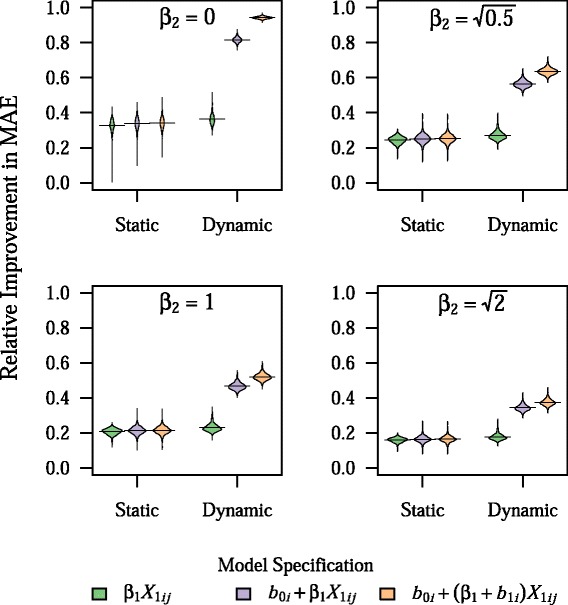
Effect of unknown patient-level predictor on model prediction accuracy. Plots show the density of values for relative improvement in MAE across 1,000 simulations, with horizontal bars representing the mean value, for different values of *β*_2_, which controls the size of the effect of the unknown patient-level predictor, *X*_2*ij*_, to the outcome, *Y*_*ij*_. Note that the relative contribution of *X*_2*ij*_ to the total variance in *Y*_*ij*_, compared to *X*_1*ij*_, is equal to *β*_2_^2^. All other parameters are fixed at their base values

When there was an unknown patient-level factor impacting the outcome (i.e. *β*_2_ ≠ 0), dynamic prediction modeling was less effective (Fig. 4). However, dynamic models still were more accurate than static models for all values of *β*_2_. Larger values of *β*_2_ were also associated with a slower rate of improvement in predictive accuracy, with 80 % of the total gains in predictive performance for the dynamic BLME model with both a random intercept and slope occurring after about 21 predictions, on average, when ${\beta}_2=\sqrt{2}$ (Additional file 1: Figure S4). Nevertheless, overall gains in prediction accuracy were still observed for clinics in the smallest quintile of clinic size, even at larger values of *β*_2_ (data not shown).

**Fig. 5:**
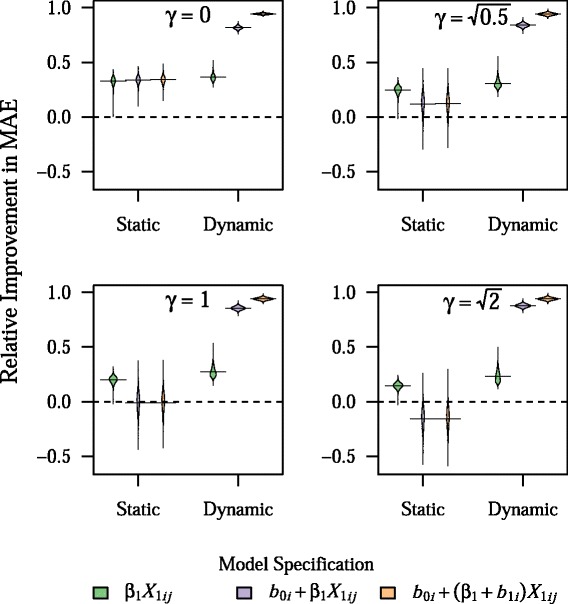
Effect of an association between clinic size and the outcome on model prediction accuracy. Plots show the density of values for relative improvement in MAE across 1,000 simulations, with horizontal bars representing the mean value, for different values of *γ*, which controls the size of the effect of scaled clinic size, *f*(*N*_*i*_), on the outcome, *Y*_*ij*_. Note that the relative contribution of *f*(*N*_*i*_) to the total variance in *Y*_*ij*_, compared to *X*_1*ij*_, is equal to *γ* ^2^. All other parameters are fixed at their base values

**Fig. 6:**
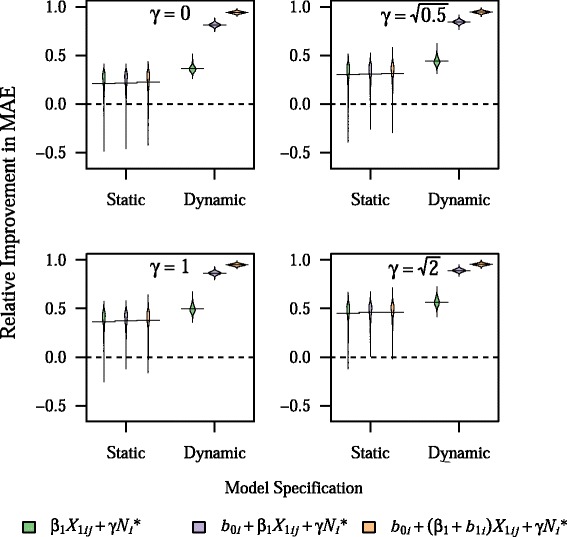
Effect of including clinic-size quintile as a fixed effect on prediction model accuracy. Plots show the density of values for relative improvement in MAE across 1,000 simulations, with horizontal bars representing the mean value, for different values of *γ*, which controls the size of the effect of scaled clinic size, *f*(*N*_*i*_), on the outcome, *Y*_*ij*_. All models include clinic-size quintile, *N*_i_^*^, as a categorical fixed effect, because *N*_i_^*^ was defined to be observed while *N*_*i*_ was defined to be unobserved. Note that the relative contribution of *f*(*N*_*i*_) to the total variance in *Y*_*ij*_, compared to *X*_1*ij*_, is equal to *γ* ^2^. All other parameters are fixed at their base values

Having the outcome be dependent on clinic size (i.e. *γ* ≠ 0) led to worse performance of static BLME models, with these models performing worse than intercept-only models at large values of *γ* (Fig. 5). However, dynamic BLME models showed no decrease in prediction accuracy with non-zero values of *γ*. Including *N*_i_^*^ as a categorical fixed effect in models led to marked improvement in static BLME models, as well as slight improvement in dynamic BLME models, on average (Fig. 6).

### Effect of varying the update interval

Results were fairly insensitive to changes in *θ*, the update interval. Even when *θ* = 5, 000, or about 12.5 % of the testing sample, prediction accuracy in dynamic BLME models was not substantially decreased (Additional file 1: Figure S5). Furthermore, prediction accuracy was consistent across all quintiles of clinic size with varying values of *θ* (Additional file 1: Figure S6). Finally, the rate of improvement in prediction accuracy showed a meaningful decrease only when *θ* = 5, 000, with about 80 % of total gains in prediction accuracy occurring on average after about 19 and 20 predictions at a given clinic for the model with a random intercept and the model with both a random intercept and random slope, respectively, in this scenario (Fig. 3).

### Computational time

**Fig. 7:**
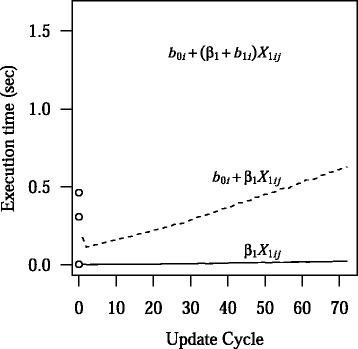
Computational time of static and dynamic models. The mean computational times in seconds for the static models are shown by the open circles. From bottom to top, the circles represent the linear model, the BLME model with a random intercept, and the BLME model with a random intercept and a random slope. The mean computational times for the dynamic linear model, BLME model with a random intercept, and BLME model with a random intercept and a random slope, are shown by the solid, dashed, and dotted lines, respectively. All parameters are fixed at their base values

Mean computational time for dynamic and static models under base parameter values are shown in Fig. 7. Static models are run once using the training sample, which had 1,276 subjects on average. After an initial reduction in computational time due to the improved efficiency from adding additional clusters, dynamic BLME models tended to have approximately linear increases in computational time with increasing number of iterations, as about 400 subjects were incorporated into the model for each round of subsequent updates.

## Discussion

In this simulation study, we sought to quantify the potential effect of dynamic prediction modeling on prediction model accuracy in the context of clustered data. Dynamic BLME models were uniformly more accurate than static models across all parameter combinations examined. Moreover, they were more accurate than static models in the context of model misspecification, and were particularly robust to misspecification of the volume-outcome relationship. As a result, it seems quite likely that the use of dynamic mixed-effects models would lead to substantial improvement in the generalizability of clinical prediction models in the context of clustered data. However, the extent of the gains in prediction accuracy from dynamic modeling was sensitive to the degree of misspecification of model fixed effects, indicating that, as with all prediction modeling, the best results will be seen when models are developed rigorously using high-quality data.

### Impact of dynamic prediction modeling

As expected, dynamic prediction modeling did not yield substantial improvement in prediction accuracy with the linear model, performing similarly to all static models, because the model did not have the flexibility to account for clinic-level variability. By contrast, dynamic BLME models were able to account for this variation, leading to improvement in predictive accuracy. The dynamic BLME model with a random intercept showed improved prediction accuracy with increasing values of *τ*_0_^2^; however, its performance deteriorated with higher values of *τ*_1_^2^. This deterioration in accuracy with larger random slopes is not surprising, because this model had no way to account for the random slopes that were present in the data. Even so, the model was able to use its random intercept to account for a large enough amount of inter-clinic variability to provide substantial and uniform improvement over static models and the dynamic linear model.

The dynamic BLME model with both a random intercept and random slope was nearly as accurate as the true model across all main parameter combinations, with a mean RI ranging from 94 to 96 %. This was because the data-generating model (Equation 7) was also based on a random intercept and random slope for most parameter combinations, and updating occurred fast enough that predictions on most individuals in the testing sample were made with a fully calibrated model. Indeed, about 80 % of the gains in prediction accuracy were seen by about the 9th patient at a given clinic, although this rate of improvement was somewhat sensitive to the magnitude of the residual error. However, even smaller clinics were still able to see benefits from dynamic prediction modeling across all of the examined parameter combinations, and the majority of predictions at large clinics were made with an accurate estimate of clinic-specific random effects. This rapid improvement in prediction accuracy was largely sustained even with higher values of *θ*, so overall prediction accuracy in the testing sample was preserved even when models were updated less frequently and using more new data per update. It should also be noted that this high level of prediction accuracy was sustained even when there was no random slope in the data-generating process (*τ*_1_^2^ = 0). Thus, there was not really much downside to having an unnecessary random slope in the dynamic BLME model, while having only a random intercept when the data-generating process included both a random intercept and a random slope led to decreased prediction accuracy.

Additionally, the variance of RI values across simulations tended to be lower in dynamic than static models. The variance in prediction accuracy decreased with each additional random effect in the model, as well. This speaks to another important feature of dynamic mixed-effects models that can be identified based on our simulation results, which is the ability to overcome sampling bias in the training sample to produce models that perform more consistently in the overall population. By contrast, in static models, the prediction accuracy was largely dependent on whether the clinics that comprised the training sample happened to be representative of the overall population. In simulations where estimates of *β*_0_ and *β*_1_ were very different from their true values due to random sampling, prediction accuracy for static models in the testing sample tended to be worse (Additional file 1: Figures S7–S9). However, dynamic models were able to overcome initial sampling bias by rapidly improving model calibration over time.

### Impact of model misspecification

Model misspecification is a common problem in clinical prediction models, as important clinical predictors are often unknown, difficult to measure, or nonlinearly related to the outcome of interest. We simulated model misspecification by including an unknown patient-level factor, *X*_2*ij*_, in the data-generating process. When this factor was allowed to influence the outcome (*β*_2_ ≠ 0), dynamic BLME models had a decrease in prediction accuracy; however, they still performed better than static models for all values of *β*_2_. More extreme values of *β*_2_ showed a similar pattern (Additional file 1: Figure S10). In short, for dynamic prediction models, it is still important to be rigorous when selecting covariates and determining their specification [26], because models that are closest to being correctly specified will still perform the best. However, the fact that dynamic mixed-effects models were more accurate in the context of model misspecification suggests that their use may be a useful strategy in the real world.

Cluster size or volume may be related to outcomes in a number of clinical scenarios, such as hospital mortality rates for acute myocardial infarction or surgical mortality rates [15, 19, 27]. While other cluster-level effects can be easily accommodated by random intercepts and slopes, volume could theoretically behave differently because it is directly related to the probability of observing the data in the first place. Larger values of *γ*, and thus larger effects of volume, led to worse performance of static BLME models, while dynamic BLME models showed no deterioration in performance. Importantly, static BLME models performed worse than static linear models when *γ* ≠ 0, unless fixed effects for clinic-size quintile (*N*_i_^*^) were included in the model. To our knowledge, this finding concerning prediction in novel clusters for static mixed-effects models has not been previously reported. Upon further examination, in static BLME models, the effect of sampling bias was actually amplified because differences due to clinic size were incorporated into the model as random effects, with greater bias in the estimated random effects covariance matrix leading to worse prediction accuracy (Additional file 1: Figures S11–S12). However, in dynamic BLME models, these initial biases rapidly diminished over time because the model was continually being calibrated to the overall population, such that the majority of predictions were unaffected by the initial biases. In essence, the volume-outcome relationship could be incorporated into cluster-specific random intercepts over time, even though this was not actually the correct specification of the data structure. As a result, inclusion of *N*_i_^*^ was required to improve the accuracy of static BLME models, but not practically necessary in the case of dynamic BLME models, at least for the cluster sizes that were included in the simulation (i.e. *N*_*i*_ ≥ 10). These results suggest that dynamic mixed-effects models can be an important tool for prediction in clinical scenarios with volume-outcome relationships, as they do not necessarily require proper specification of this relationship to yield dramatic improvements in prediction accuracy.

We also conducted sensitivity analyses where both the known and unknown patient-level factors, *X*_1*ij*_ and *X*_2*ij*_, had a non-linear relationship with the outcome by adding squared terms to the data-generating model (Additional file 1: Figures S13–S14). The results of these sensitivity analyses were similar, in that the gains of prediction accuracy from using dynamic BLME models were reduced in scenarios with greater degrees of model misspecification, although for both static and dynamic models the magnitude of the reduction in RI from misspecification of non-linear terms was somewhat larger than seen from misspecification of linear terms. However, even in the case of misspecification of non-linear relationships, there was no scenario identified in which dynamic BLME models were less accurate than static BLME models.

Interestingly, in cases of extreme model misspecification, there seemed to be a pattern of the dynamic BLME model with both a random intercept and a random slope having slightly worse prediction accuracy than the dynamic BLME model with only a random intercept. This result may suggest that dynamic BLME models with more complexity or greater degrees of freedom may perform slightly worse in situations of extreme model misspecification, perhaps because these models are somewhat more likely to suffer from overfitting of noise in the data. Thus, using more conservative dynamic models may be prudent in situations where extreme misspecification is more likely. However, the large gains in accuracy from model updating always exceeded the minor effects of overfitting, making even complex dynamic models superior to static models.

### Impact of the update interval

Previous research on dynamic prediction models have not examined whether the frequency with which the model is updated would impact the expected gains in prediction accuracy. In many clinical scenarios, updating frequency could be limited by computational constraints or logistical challenges related to data collection, as well as by time lags between when predictions are made and when the outcomes actually occur. Fortunately, our results show that gains in prediction accuracy seen with dynamic mixed-effects models are robust to less frequent updating intervals, with only minor reductions in prediction accuracy at very high values of *θ*. As a result, dynamic mixed-effects models should be feasible in situations where real world constraints limit the frequency of model updating.

### Challenges to using dynamic prediction modeling in practice

Implementation of dynamic mixed-effects models in practice will likely involve many logistical and analytical challenges. Ideally, prediction models would be integrated into electronic health record systems, so they will be able to automatically extract covariate data to make an initial prediction, and then automatically extract outcome data to use for model updating. Furthermore, in order to accommodate heterogeneities across sites, the electronic health record will need to either be standardized across all of the sites, or be compatible enough to allow for communication of data. Additionally, the data storage and security requirements for large amounts of data across multiple sites will likely be quite complex. Certain analytic strategies—such as Bayesian dynamic regression, where posterior distributions are estimated from dynamic priors in a fully online fashion [14]—could greatly reduce the data storage requirements, and, accordingly, the data security concerns. These analytic strategies may also help reduce the computational burden of running dynamic models on increasingly large amounts of data, as well. However, more simulation work is needed to determine the trade-offs in prediction accuracy that might accompany this estimation approach under certain scenarios. Finally, there will need to be a concerted effort to communicate the effectiveness of this approach to the clinical community in order to foster the necessary level of trust to overcome initial financial and logistical hurdles.

The analytic challenges involved with dynamic prediction modeling are also likely to be quite complex. Missing data, both for covariates and outcomes, will be an important issue to resolve, because standard methods, such as multiple imputation [28, 29], may be difficult to implement in the context of a dynamic system. As a result, efforts to jointly model the updating process along with the prediction model itself, analogous to methods for jointly modeling longitudinal and competing risks data [30], may be required. Alternatively, use of missing indicators may be of greater use than with standard models [31], because these parameters would be allowed to calibrate to the population over time. However, further studies are needed to answer these questions empirically.

### Study limitations

Although our simulation was based on a hypothetical predictor and outcome variable, we tried wherever possible to mimic situations that might occur when developing and utilizing a typical clinical prediction model. For instance, we used a log-normal distribution for clinic size, so that there would be a larger number of small clinics than large clinics, and we generated the training sample to be similar in size and composition to a large multi-center cohort study. We also excluded some patients from contributing data to dynamic models, to reflect the loss to follow-up that might occur in clinical practice. Finally, we examined scenarios where the model was not correctly specified, which are likely to occur in real-world applications.

Despite these efforts, there were still a number of limitations to our model. For instance, we did not examine scenarios where heterogeneities across clinics were not normally distributed. It is possible that standard BLME models might not perform as well in this scenario, leading to a model that was less calibrated to local conditions, even after updating. However, research studying the impact of misspecified parameterization of random effects on prediction accuracy suggests that the standard multivariate normal assumptions should be reasonably robust [32]. Additionally, we assumed in our simulation that outcome data that were not available for updating were missing completely at random, which may not hold in practice. Future studies are needed to determine whether the prediction accuracy of dynamic prediction models will be worsened in scenarios where the probability of obtaining outcome data for updating is dependent on model covariates or, especially, the outcome.

Furthermore, we only tested the simple case of a normally distributed, linear outcome. It is possible that the relative rate of improvement in model accuracy for dynamic models could be different for other types of data, such as binary or count data, due to differences in the relative efficiency of the models involved. Because GLMMs are asymptotically consistent regardless of the link function or error distribution used, we would expect that dynamic mixed-effects models would also show overall improvement compared to static models regardless of the type of data under consideration, since the process of model updating allows for the accumulation of additional data. More research, however, will be needed to formally test the relative performance of dynamic mixed-effects modeling under various conditions for other distributions in the exponential family, or even non-exponential data, such as survival data.

We attempted to cover a reasonable range of parameter values in our analysis, including some parameter values that reflect more extreme cases of model misspecification; however, it is possible that our results will not extrapolate to values outside of the tested ranges. For instance, cluster size was rarely less than ten individuals for our simulation; however, there is empirical evidence to suggest that mixed-effects models may perform poorly on such very small clusters when there is a strong volume-outcome relationship [19]. As a result, we would encourage caution when utilizing dynamic mixed-effects models in clusters with less than ten individuals when there is a known strong volume-outcome relationship. Additionally, to reduce computational burdens, we focused on a simplistic model: a single continuous predictor and a continuous outcome. Clearly, clinical prediction models in the real world will have multiple covariates, and many will have more complex outcomes. The exact gains in prediction accuracy from dynamic prediction modeling will likely vary depending on the particular structure of the data in question, with more complex models likely requiring more time and more data to become fully calibrated. Future research is needed to better characterize the performance of dynamic mixed-effects models as a function of model complexity. Finally, dynamic prediction modeling in practice will have to deal with a lag between when predictions are made and when outcomes are observed. For instance, in models predicting five-year survival in cancer, it could be years before outcomes are obtained to be included for model updating. It is possible that long lag periods relative to the frequency of updating will decrease the rate at which prediction accuracy improves. As a result, dynamic mixed-effects models may be less useful for outcomes with long lag times, especially at smaller clinics or in rapidly changing populations. We attempted to assess the sensitivity of our results to long lag times by varying the update interval, *θ*, and large improvements in prediction accuracy with dynamic BLME models were still seen even at the highest values of *θ*. Even with these positive results, though, the exact effect of time lags on the performance of dynamic prediction models will need to be formally addressed in future research.

## Conclusions

In conclusion, use of dynamic mixed-effects models led to more accurate predictions in the overall population compared with static prediction models. The extent of the improvement in prediction accuracy that was observed depended on the relative impact of fixed and random effects on the outcome as well as the degree of model misspecification. Nonetheless, dynamic mixed-effects models were uniformly superior to static models as well as dynamic models with only fixed effects. Gains in prediction accuracy tended to occur rapidly, leading to improvements at small clinics as well as large clinics. Dynamic mixed-effects models were also particularly robust to misspecification of the volume-outcome relationship as well as to variation in the update interval. While there are many logistical and analytical questions to resolve, dynamic mixed-effects models appear to be a useful approach for improving the accuracy and generalizability of clinical prediction models in the context of clustered data.

## Additional file

Additional file 1: **Supplementary Material.** **Figure S1.** Effect of the size of the random intercept and random slope on clinic-level clustering. **Figure S2.** Effect of the size of the residual error on the rate of improvement in prediction accuracy at a given clinic. **Figure S3.** Effect of the size of the residual error on model prediction accuracy. **Figure S4.** Effect of unknown patient-level predictor on the rate of improvement in prediction accuracy at a given clinic. **Figure S5.** Effect of the update interval on model prediction accuracy. **Figure S6.** Effect of the update interval on model prediction accuracy by clinic-size quintile. **Figure S7.** Relationship between bias in estimated model coefficients and prediction accuracy for the linear model. **Figure S8.** Relationship between bias in estimated model coefficients and prediction accuracy for the BLME model with random intercept. **Figure S9.** Relationship between bias in estimated model coefficients and prediction accuracy for the BLME model with random intercept and slope. **Figure S10.** Effect of extreme values of *β*_2_ on model prediction accuracy. **Figure S11.** Relationship between bias in estimated model coefficients and prediction accuracy for the BLME model with random intercept, with clinic size influencing the outcome. **Figure S12.** Relationship between bias in estimated model coefficients and prediction accuracy for the BLME model with random intercept and slope, with clinic size influencing the outcome. **Figure S13.** Effect of a non-linear relationship in the known patient-level predictor on model prediction accuracy. **Figure S14.** Effect of a non-linear relationship in the unknown patient-level predictor on model prediction accuracy. (PDF 3602 kb)

## Abbreviations

BLME: Bayesian linear mixed-effects
GLMM: generalized linear mixed-effects model
MAE: mean absolute error
RI: relative improvement in mean absolute error
