# Supplementary material for: The prediction accuracy of dynamic mixed-effects models in clustered data
Source: BioData Min. 2016 Jan 27;9:5. doi: 10.1186/s13040-016-0084-6 (PMC4728760; doi:10.1186/s13040-016-0084-6)

The prediction accuracy of dynamic mixed-effects models in clustered data

Brian S. Finkelman, Benjamin French, and Stephen E. Kimmel

Supplementary Material

Figure S1. Effect of the size of the random intercept and random slope on clinic-level clustering. Each point represents an individual patient at one of the 20 clinics in the training sample for a single simulation run. Lines represent the actual relationship between X_{1ij} and Y_{ij} at each clinic. Each figure shows a different combination of values for τ_0^2, which represents the variance in the random intercept in the population, and τ_1^2, which represents the variance in the random slope in the population. All other parameters are fixed at their base values. The center figure represents the base parameter combination. Note that increasing the value of τ_0^2 leads to increased vertical displacement of clinic-specific slopes, while increasing the value of τ_1^2 leads to an increased fanning pattern.

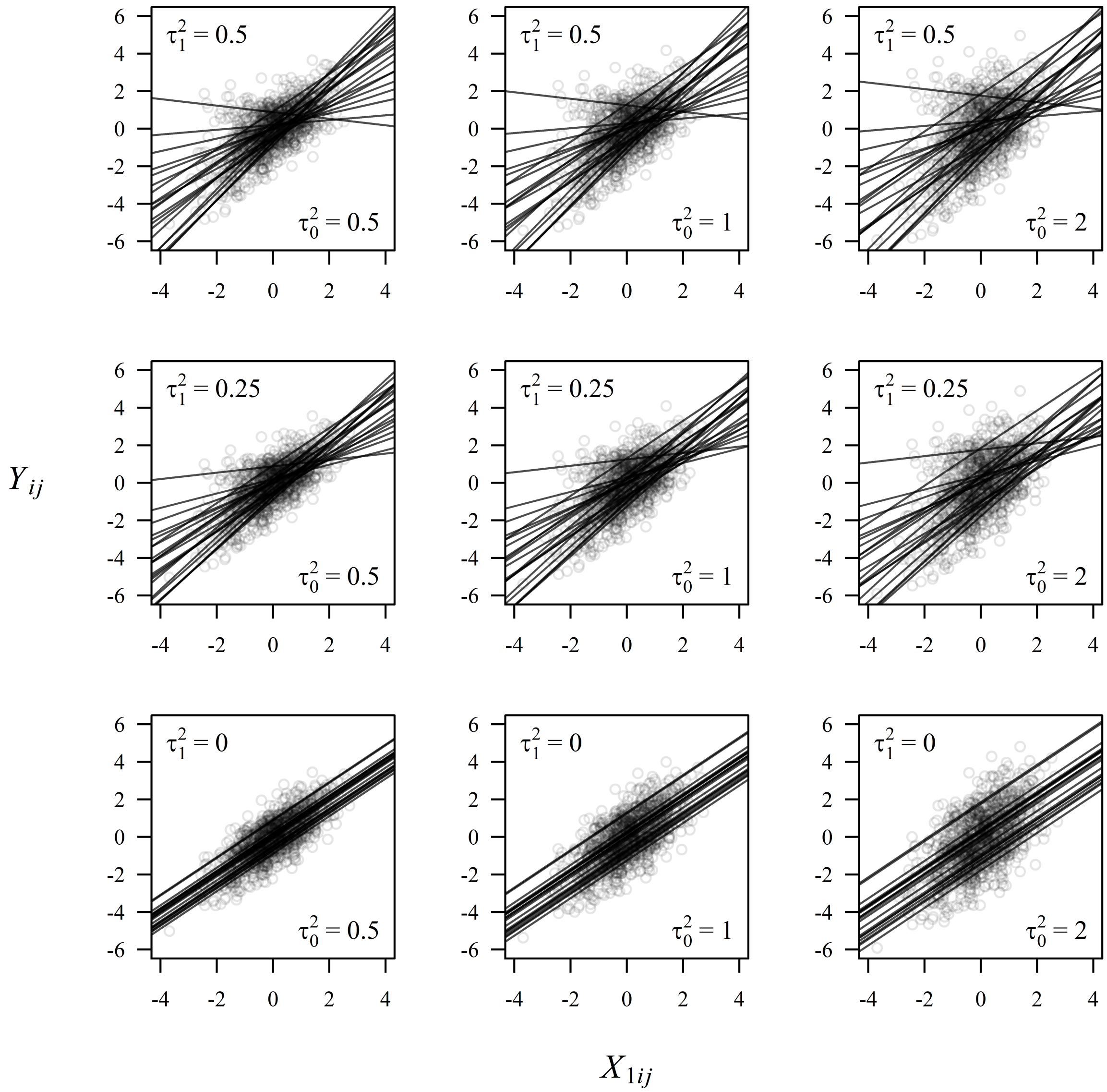

Figure S2. Effect of the size of the residual error on the rate of improvement in prediction accuracy at a given clinic. This plot shows the mean relative improvement in MAE for prediction j at clinic i, across 1,000 simulations for different values of α, which is equal to the relative size of the residual error compared to the overall variance in the outcome, $\sigma_\varepsilon^2/\sigma_Y^2$. The vertical dashed line indicates the point at which 80% of the total gains in prediction accuracy have been achieved for the dynamic BLME model with a random intercept and random slope. Note that the base value of α is 0.2, and all other parameters are fixed at their base values.

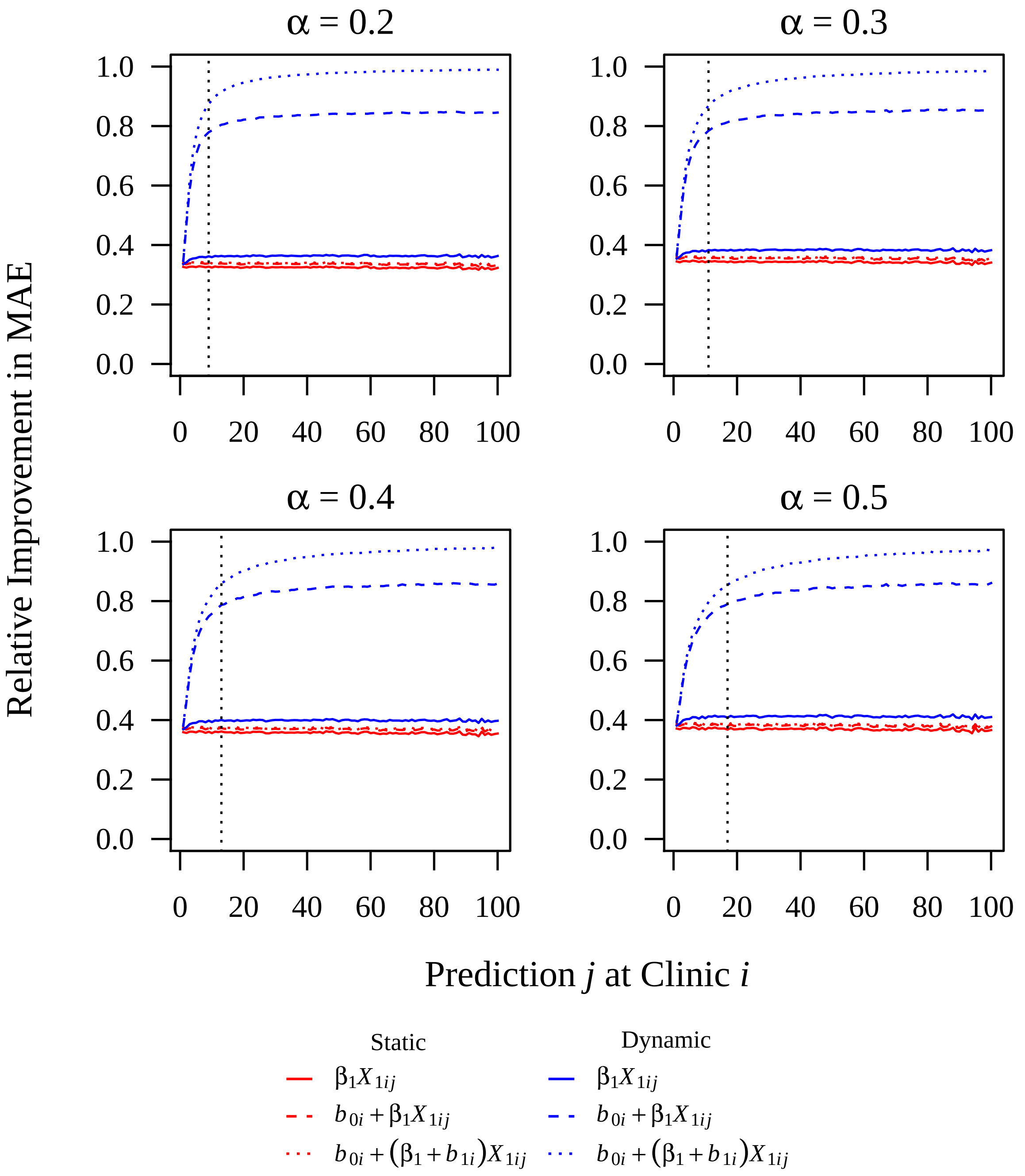

Figure S3. Effect of the size of the residual error on model prediction accuracy. Plots show the density of values for relative improvement in MAE across 1,000 simulations, with horizontal bars representing the mean value, for different values of α, which is equal to the relative size of the residual error compared to the overall variance in the outcome, $\sigma_\varepsilon^2/\sigma_Y^2$. All other parameters are fixed at their base values.

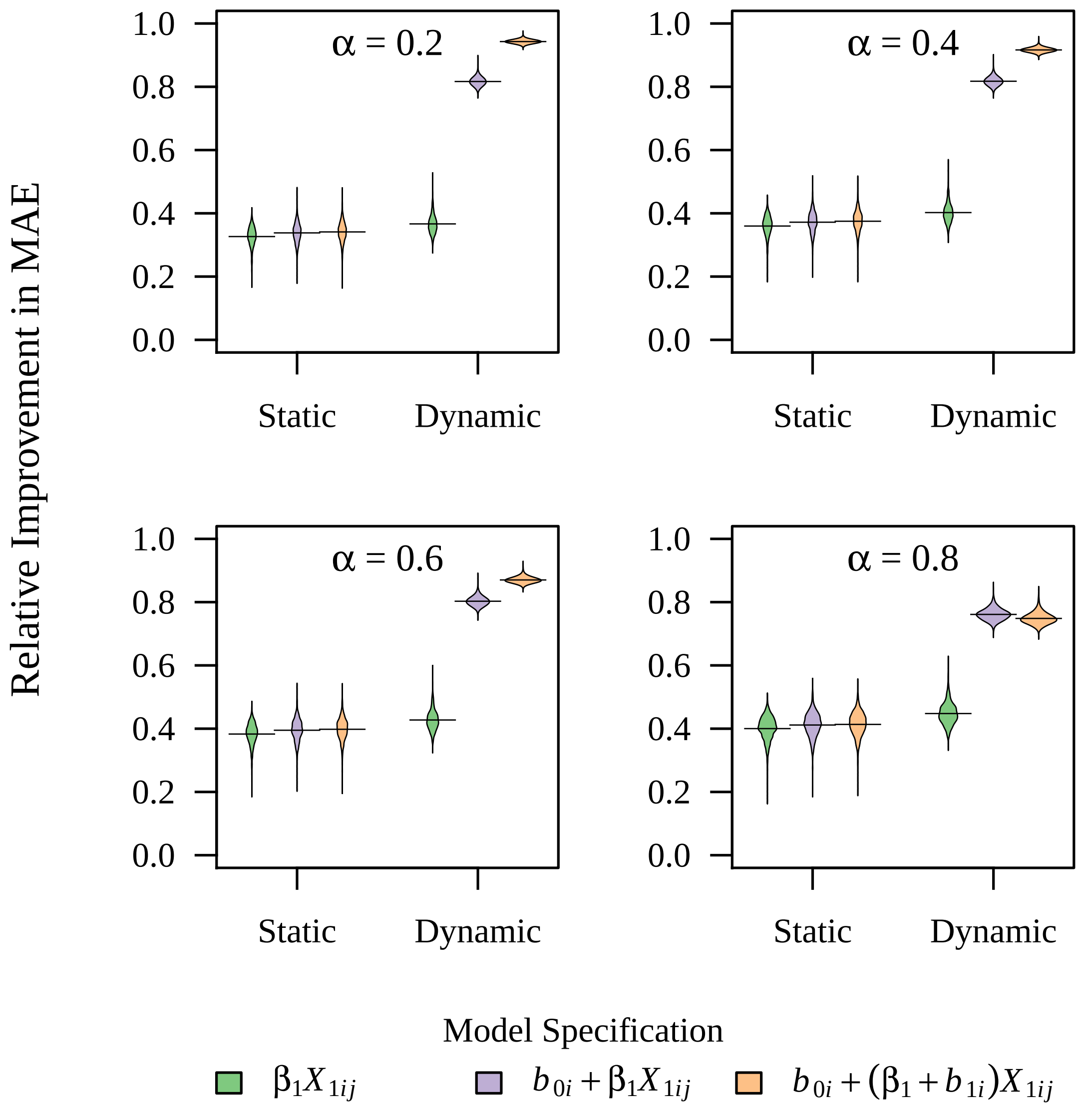

Figure S4. Effect of unknown patient-level predictor on the rate of improvement in prediction accuracy at a given clinic. This plot shows the mean relative improvement in MAE for prediction j at clinic i, across 1,000 simulations for different values of the relative size of β_2, which controls the size of the effect of the unknown patient-level predictor, X_{2ij}, to the outcome, Y_{ij}. The vertical dashed line indicates the point at which 80% of the total gains in prediction accuracy have been achieved for the dynamic BLME model with a random intercept and random slope. Note that the relative contribution of X_{2ij} to the total variance in Y_{ij}, compared to X_{1ij}, is equal to β_2^2. All other parameters are fixed at their base values.

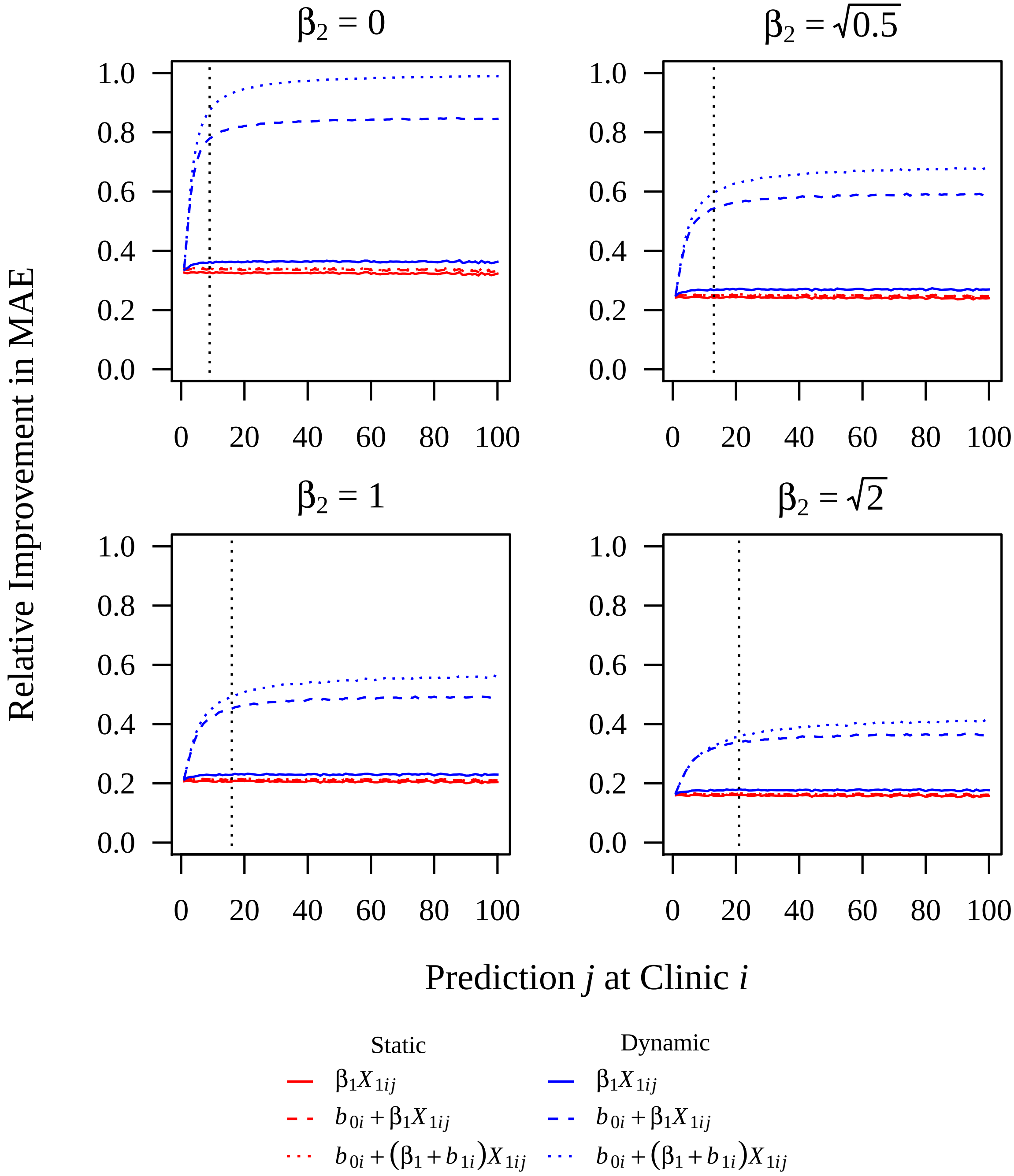

Figure S5. Effect of the update interval on model prediction accuracy. Plots show the density of values for relative improvement in MAE across 1,000 simulations, with horizontal bars representing the mean value, for different values of the update interval, θ. All other parameters are fixed at their base values.

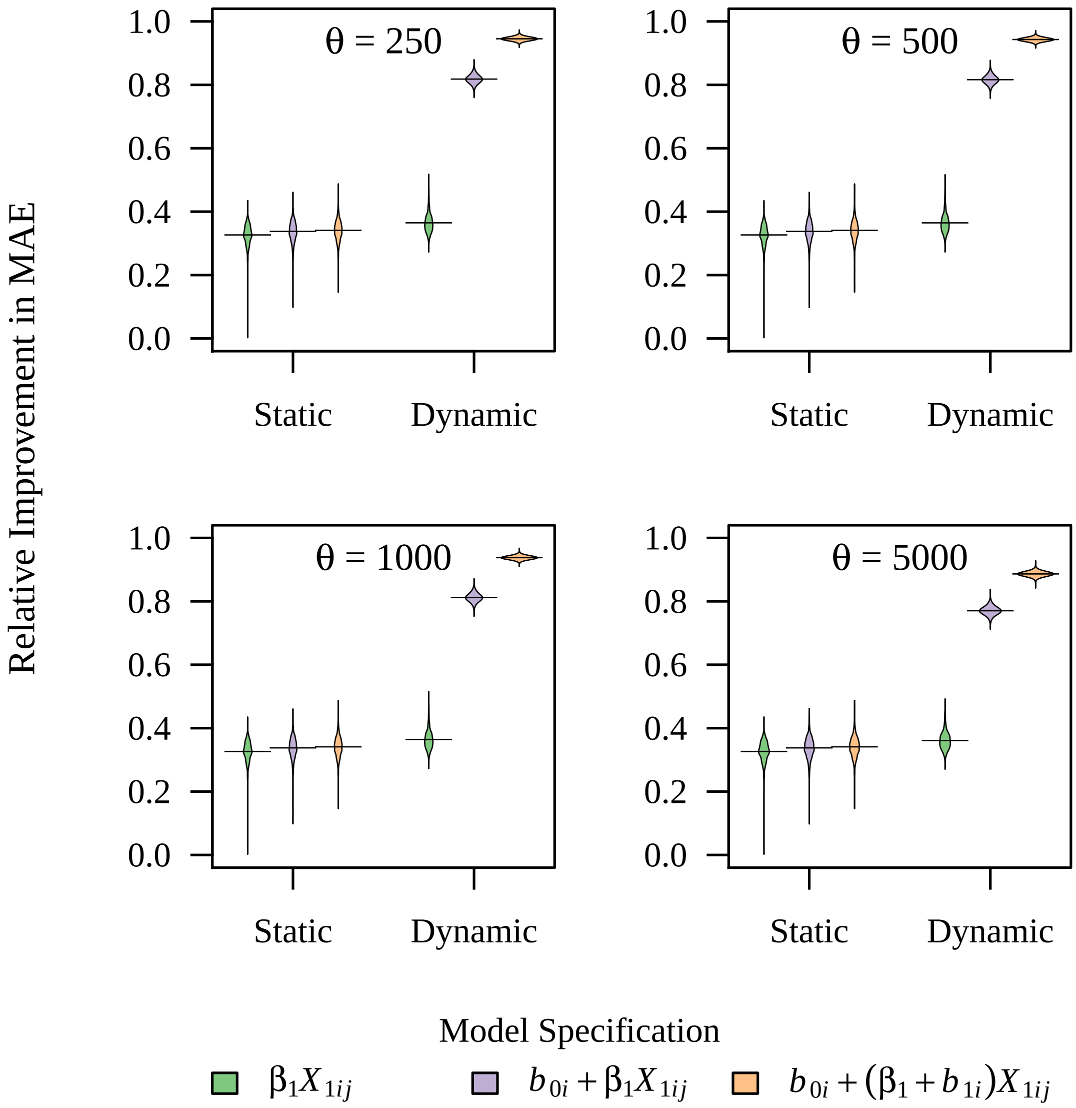

Figure S6. Effect of the update interval on model prediction accuracy by clinic-size quintile. Plots show the density of values for relative improvement in MAE across 1,000 simulations, with horizontal bars representing the mean value, for different values of the update interval, θ. The base value of θ is 500, and all other parameters are fixed at their base values.

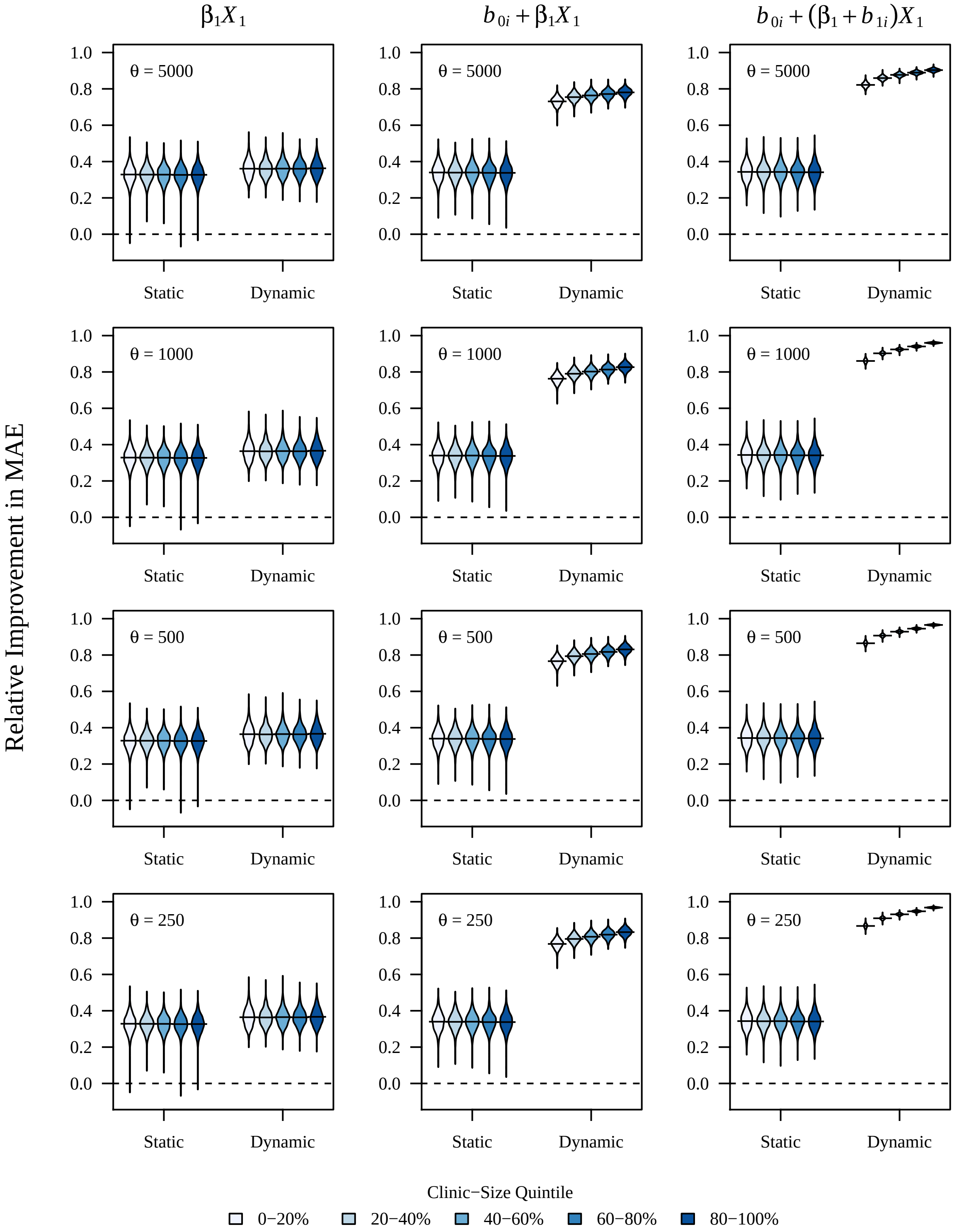

Figure S7. Relationship between bias in estimated model coefficients and prediction accuracy for the linear model. Each point represents one of 1,000 total simulations for the base parameter combination, and best fit lines are shown in red. The left figure shows the bias in the estimated intercept from the training sample compared to the true value in the overall population, while the right figure shows this bias for the estimated slope.

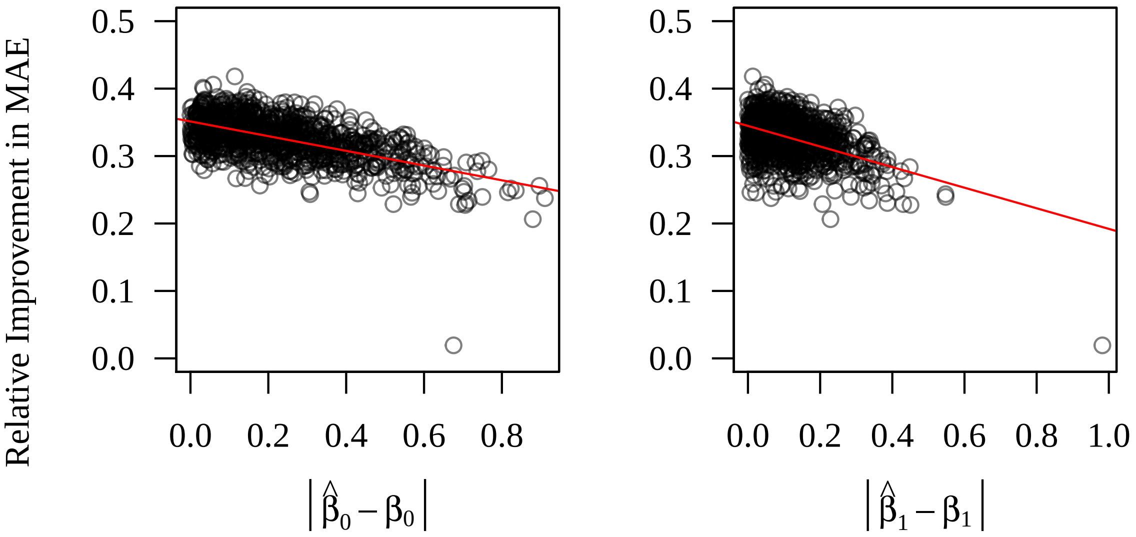

Figure S8. Relationship between bias in estimated model coefficients and prediction accuracy for the BLME model with random intercept. Each point represents one of 1,000 total simulations for the base parameter combination, and best fit lines are shown in red. The upper left figure shows the bias in the estimated intercept from the training sample compared to the true value in the overall population, the upper right figure shows this bias for the estimated slope, and the bottom figure shows this bias for the estimated variance of the random intercepts.

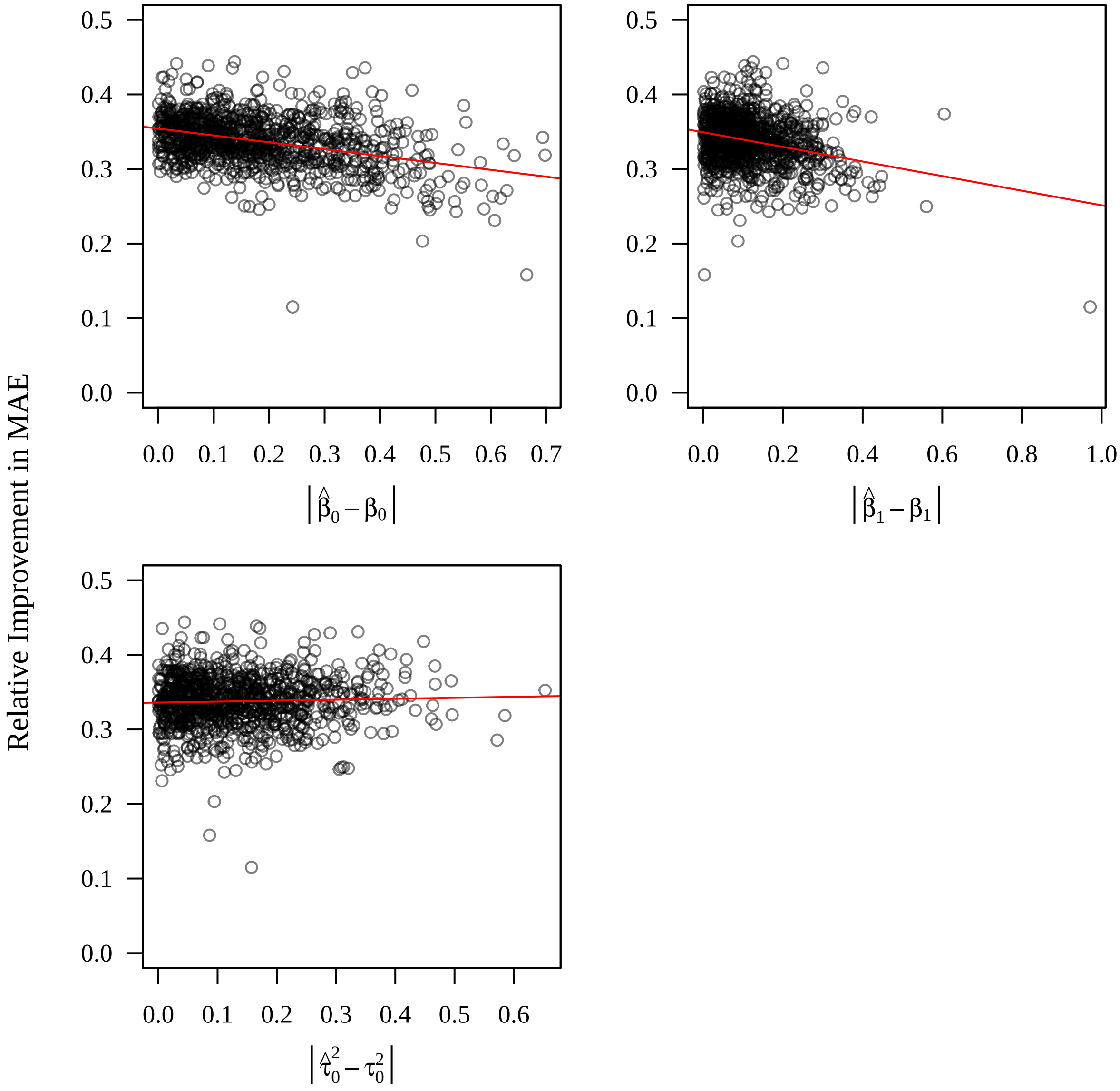

Figure S9. Relationship between bias in estimated model coefficients and prediction accuracy for the BLME model with random intercept and slope. Each point represents one of 1,000 total simulations for the base parameter combination, and best fit lines are shown in red. Starting from the top left figure and moving in clockwise fashion, the panels show the bias in the estimated intercept, slope, variance in the random slopes, and variance in the random intercepts, as compared to the true value in the overall population.

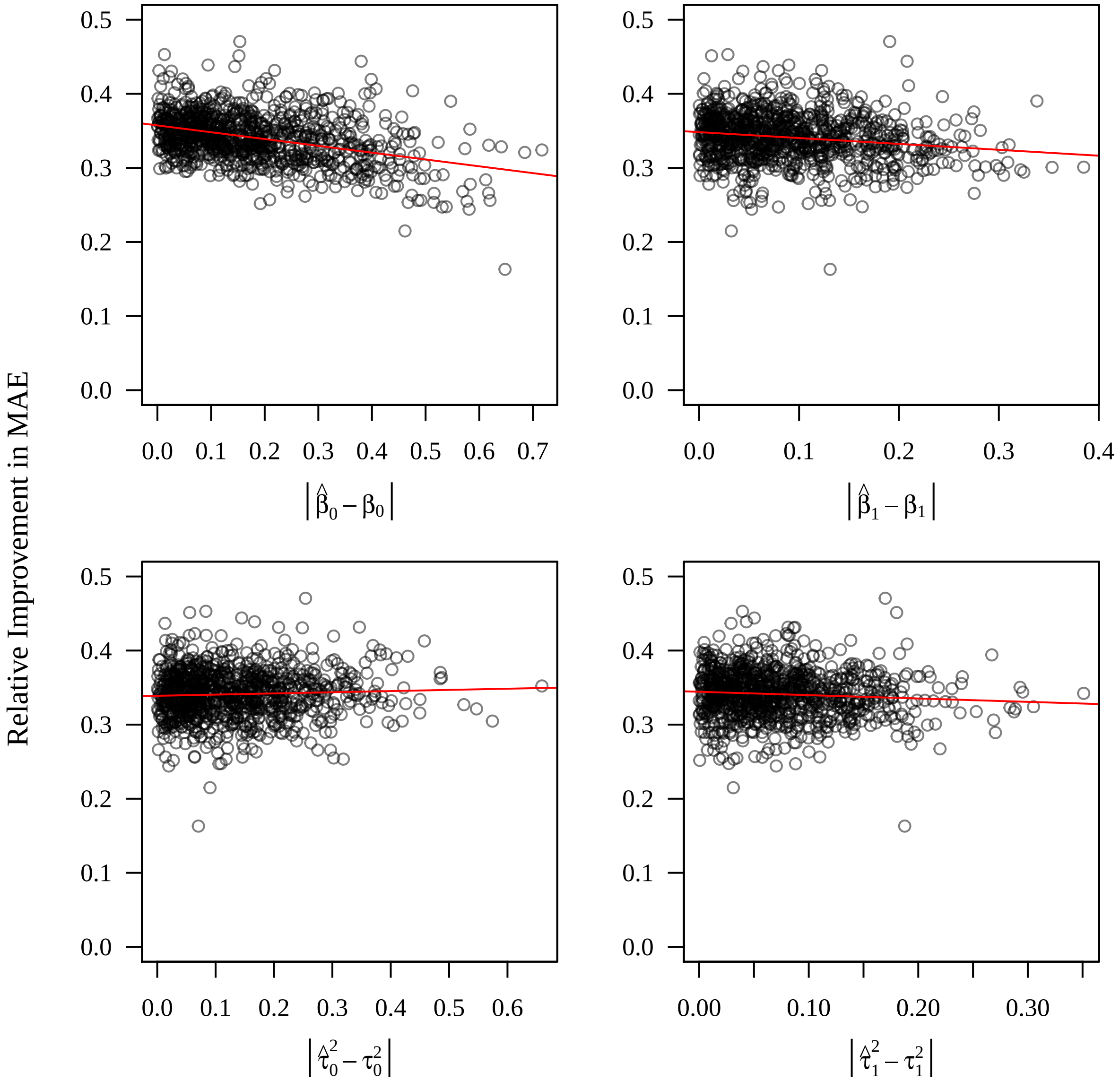

Figure S10. Effect of extreme values of β_2 on model prediction accuracy. Plots show the density of values for relative improvement in MAE across 1,000 simulations, with horizontal bars representing the mean value, for different values of β_2, which controls the size of the effect of the unknown patient-level predictor, X_{2ij}, to the outcome, Y_{ij}. Note that the relative contribution of X_{2ij} to the total variance in Y_{ij}, compared to X_{1ij}, is equal to β_2^2. All other parameters are fixed at their base values.

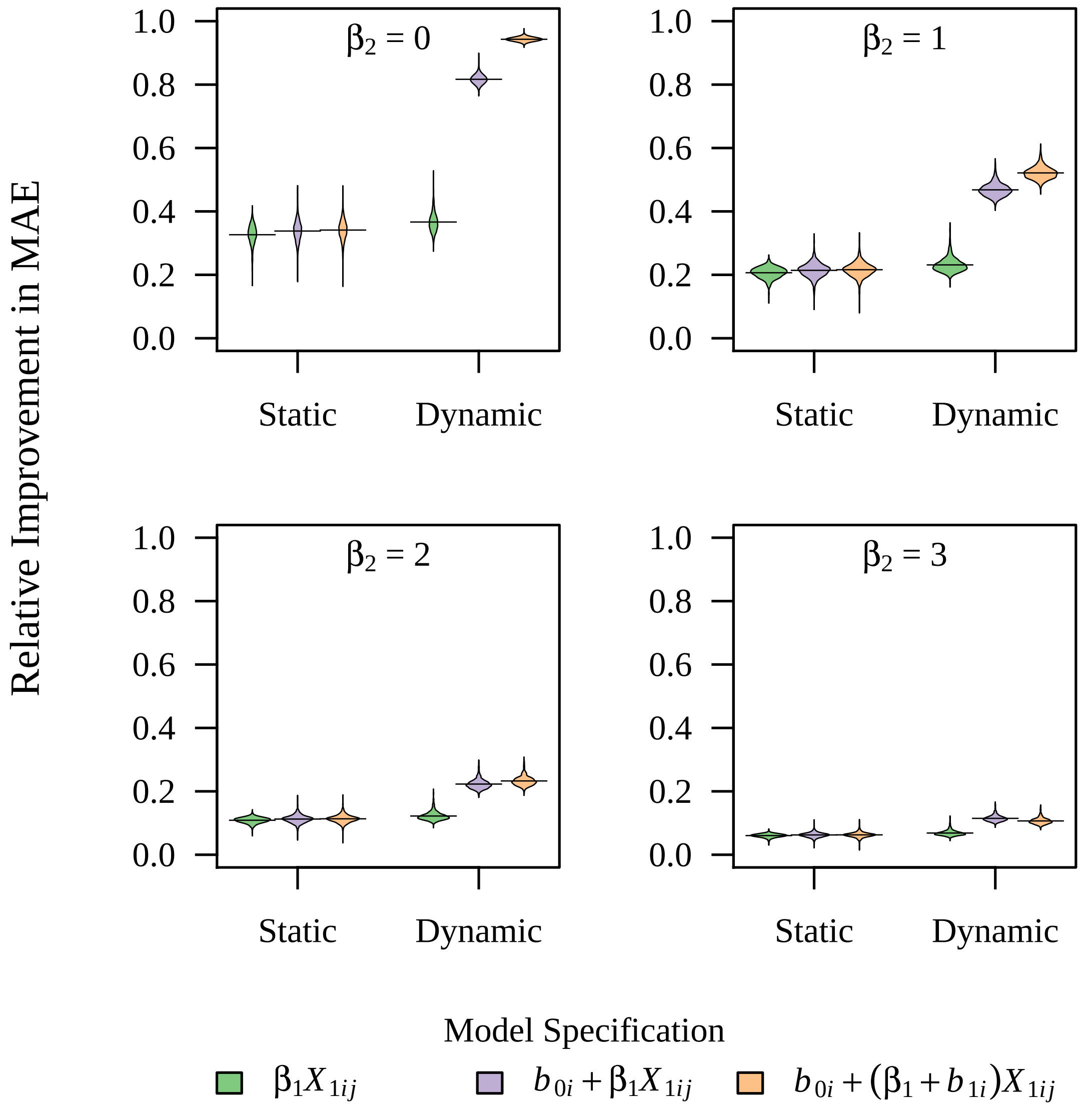

Figure S11. Relationship between bias in estimated model coefficients and prediction accuracy for the BLME model with random intercept, with clinic size influencing the outcome. Each point represents one of 1,000 total simulations with $\gamma = \sqrt{2}$, the largest value tested, and best fit lines are shown in red. The upper left figure shows the bias in the estimated intercept from the training sample compared to the true value in the overall population, the upper right figure shows this bias for the estimated slope, and the bottom figure shows this bias for the estimated variance of the random intercepts.

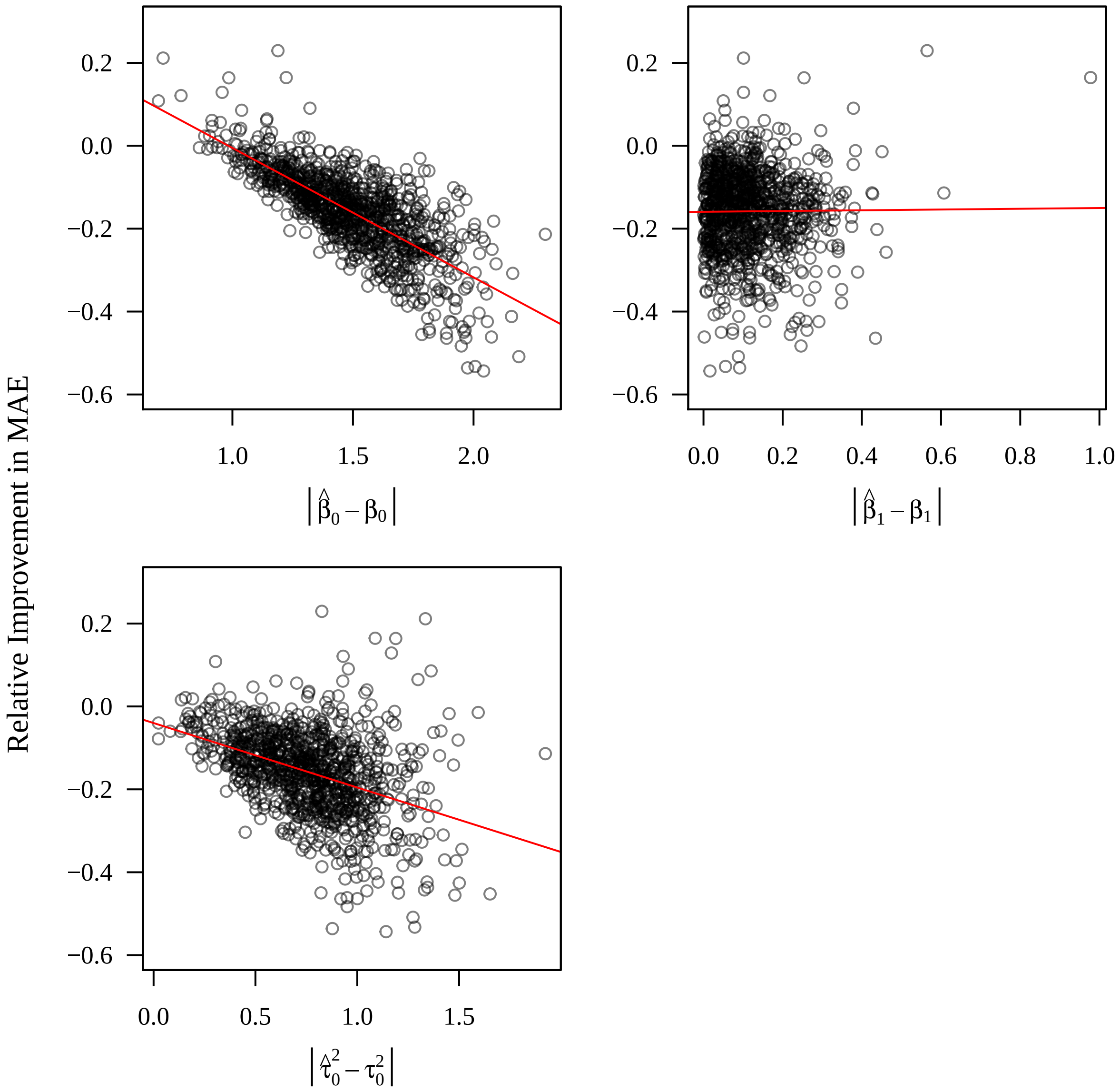

Figure S12. Relationship between bias in estimated model coefficients and prediction accuracy for the BLME model with random intercept and slope, with clinic size influencing the outcome. Each point represents one of 1,000 total simulations with $\gamma = \sqrt{2}$, the largest value tested, and best fit lines are shown in red. Starting from the top left figure and moving in clockwise fashion, the panels show the bias in the estimated intercept, slope, variance in the random slopes, and variance in the random intercepts, as compared to the true value in the overall population.

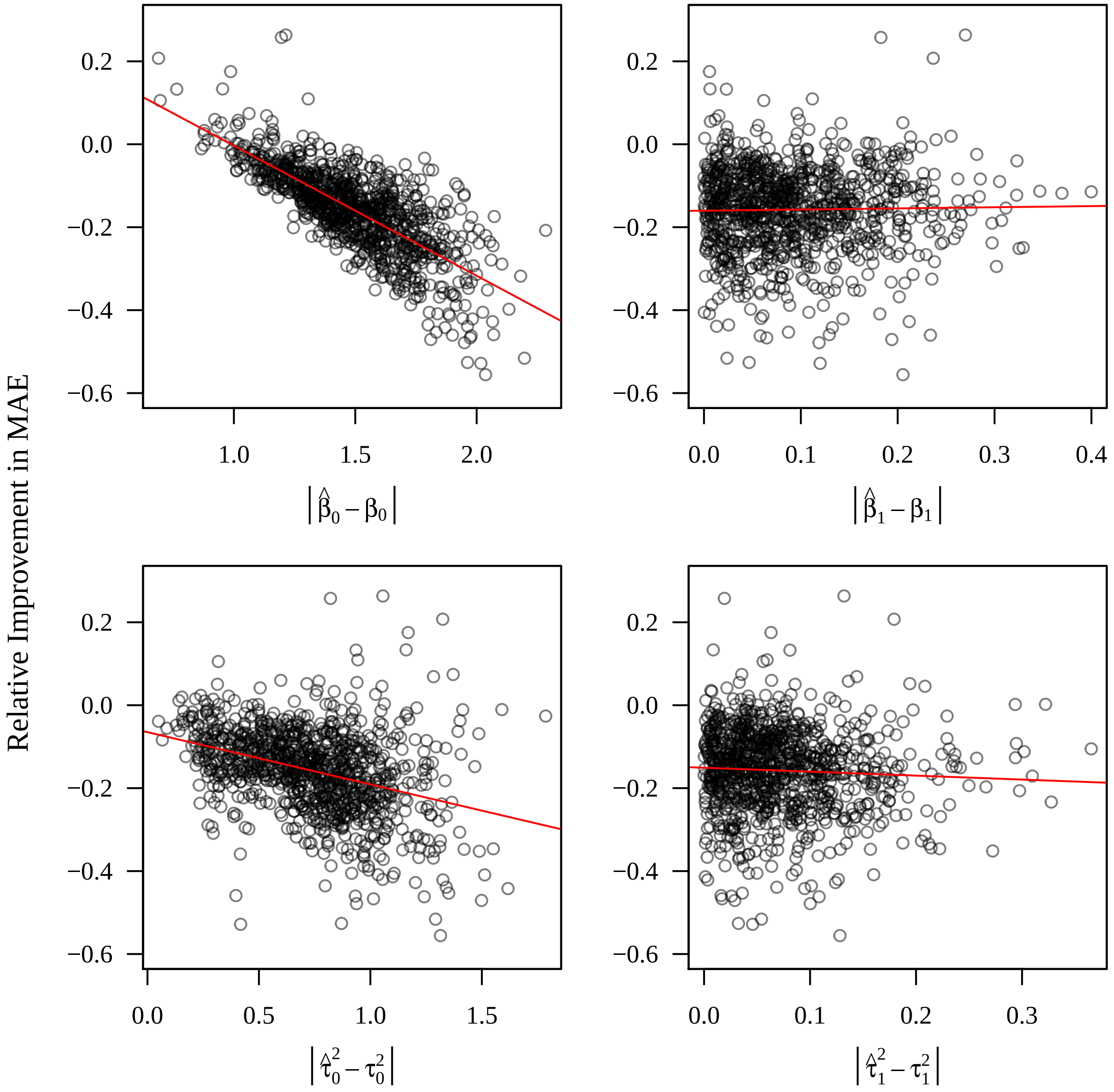

Figure S13. Effect of a non-linear relationship in the known patient-level predictor on model prediction accuracy. For these simulations, a squared term, $\Psi_{\beta_1}(\beta_1 + b_{1i}) X_{1ij}^2$, was added to the data-generating model, shown in Equation 7. Plots show the density of values for relative improvement in MAE across 1,000 simulations, with horizontal bars representing the mean value, for different values of Ψ_{β_1}, which controls the relative impact of X_{1ij}^2 and X_{1ij} on the outcome. Note that the relative contribution of X_{1ij}^2 to the total variance in Y_{ij}, compared to X_{1ij}, is equal to $\Psi_{\beta_1}^2$. All other parameters are fixed at their base values.

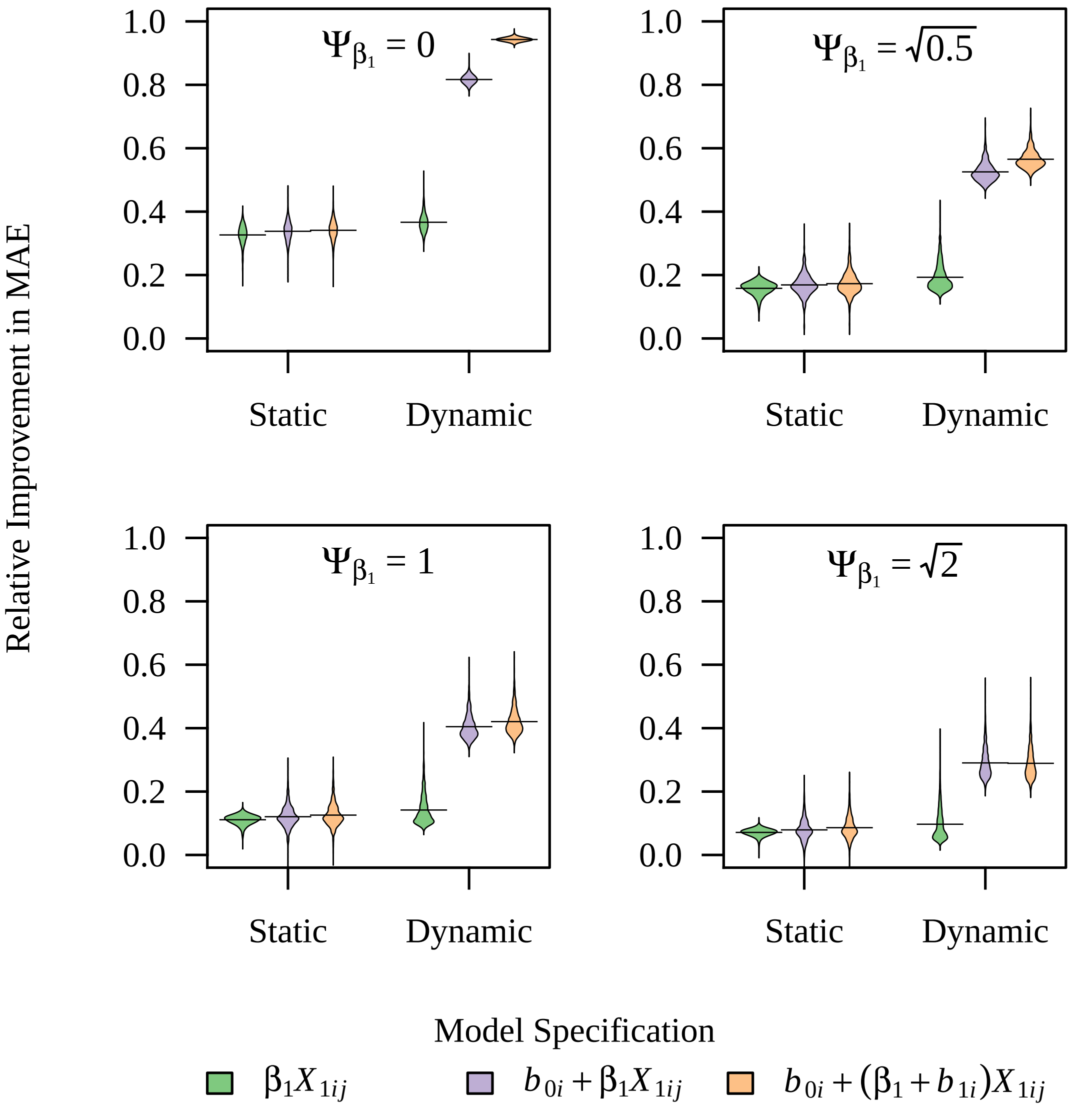

Figure S14. Effect of a non-linear relationship in the unknown patient-level predictor on model prediction accuracy. For these simulations, a squared term, $\Psi_{\beta_2}(\beta_2 + b_{2i}) X_{2ij}^2$, was added to the data-generating model, shown in Equation 7. Plots show the density of values for relative improvement in MAE across 1,000 simulations, with horizontal bars representing the mean value, for different values of Ψ_{β_2}, which controls the relative impact of X_{2ij}^2 and X_{2ij} on the outcome. Note that the relative contribution of X_{2ij}^2 to the total variance in Y_{ij}, compared to X_{2ij} or X_{1ij}, is equal to $\Psi_{\beta_1}^2$. For these simulations, β_2 is fixed at 1, and all other parameters are fixed at their base values.

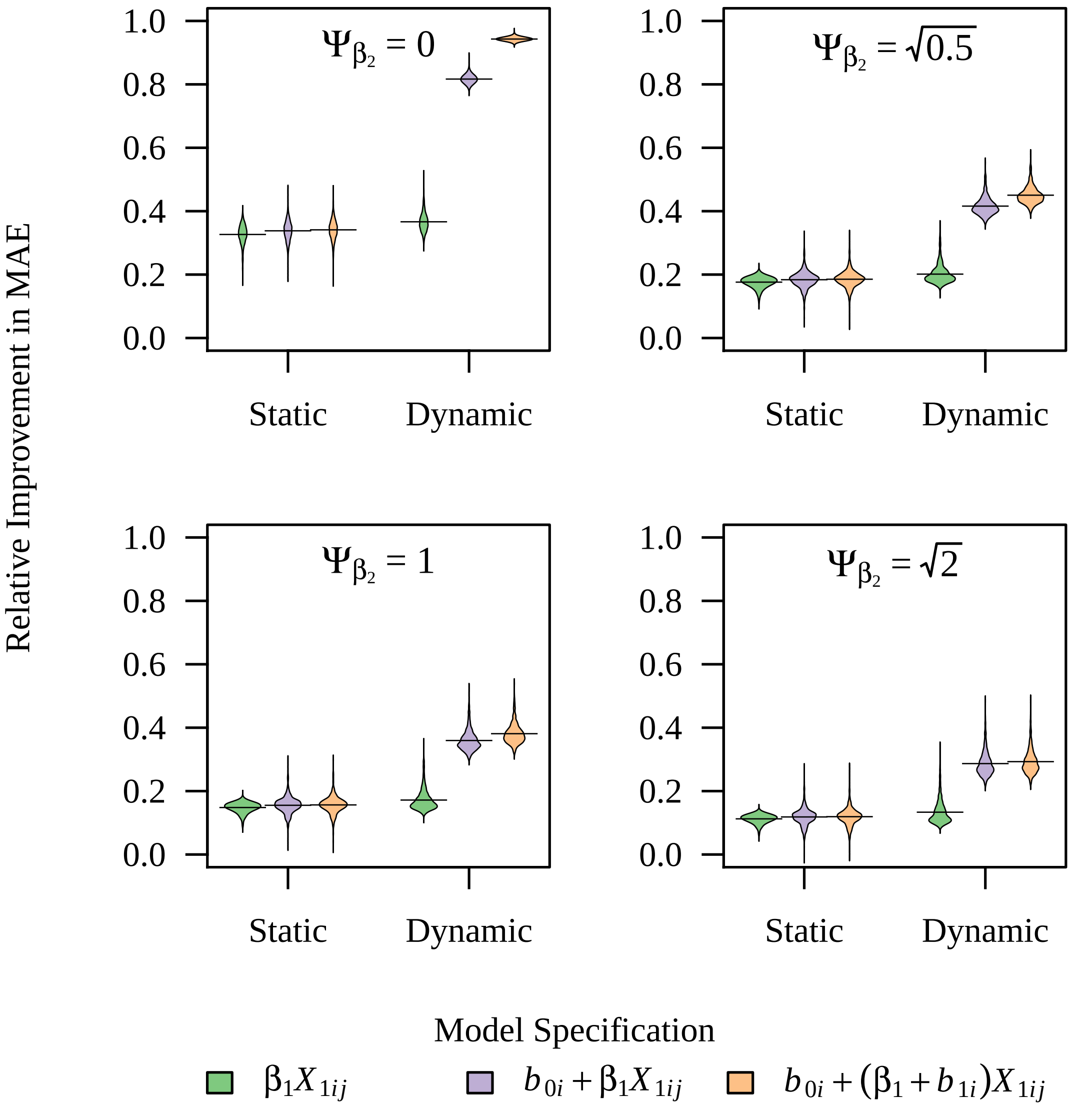

Supplement: Supplementary file 1 — Supplementary Material. Figure S1. Effect of the size of the random intercept and random slope on clinic-level clustering. Figure S2. Effect of the size of the residual error on the rate of improvement in prediction accuracy at a given clinic. Figure S3. Effect of the size of the residual error on model prediction accuracy. Figure S4. Effect of unknown patient-level predictor on the rate of improvement in prediction accuracy at a given clinic. Figure S5. Effect of the update interval on model prediction accuracy. Figure S6. Effect of the update interval on model prediction accuracy by clinic-size quintile. Figure S7. Relationship between bias in estimated model coefficients and prediction accuracy for the linear model. Figure S8. Relationship between bias in estimated model coefficients and prediction accuracy for the BLME model with random intercept. Figure S9. Relationship between bias in estimated model coefficients and prediction accuracy for the BLME model with random intercept and slope. Figure S10. Effect of extreme values of β 2 on model prediction accuracy. Figure S11. Relationship between bias in estimated model coefficients and prediction accuracy for the BLME model with random intercept, with clinic size influencing the outcome. Figure S12. Relationship between bias in estimated model coefficients and prediction accuracy for the BLME model with random intercept and slope, with clinic size influencing the outcome. Figure S13. Effect of a non-linear relationship in the known patient-level predictor on model prediction accuracy. Figure S14. Effect of a non-linear relationship in the unknown patient-level predictor on model prediction accuracy. (PDF 3602 kb) [file 13040_2016_84_MOESM1_ESM.pdf]
